# Effect of Body Composition and Age on the Subjective and Quantitative Ultrasound Appearance of the Dogs’ Pancreas

**DOI:** 10.1111/vru.70208

**Published:** 2026-07-15

**Authors:** Robert B. S. Turner, Simon M. Firestone, Frank R. Dunshea, Caroline Mansfield

**Affiliations:** ^1^ Melbourne Veterinary School, Faculty of Science University of Melbourne Werribee Victoria Australia; ^2^ School of Agriculture, Food and Ecosystem Sciences, Faculty of Sciences University of Melbourne Parkville Victoria Australia; ^3^ School of Biology, Faculty of Biological Science The University of Leeds Leeds UK; ^4^ Department of Small Animal Clinical Sciences, College of Veterinary Medicine Michigan State University East Lansing Michigan USA

**Keywords:** dog, pancreas, quantitative image analysis, ultrasonography

## Abstract

Ultrasound assessment of the pancreas in dogs is limited by operator subjectivity and technical variability, particularly when classifying echotexture and echogenicity. This prospective study evaluated quantitative ultrasonographic features of the pancreas in dogs and examined their associations with subjective ultrasonographic features, age, body composition, and technical factors. Seventy‐two ultrasounds from client‐owned dogs undergoing abdominal imaging for diverse clinical indications between 2016 and 2019 were included. Subjective echogenicity and echotexture were assessed, pancreatic and abdominal adipose thicknesses measured, and quantitative intensity and texture features extracted from regions of interest using image‐analysis software. A subset of cases was evaluated by 104 veterinary radiologists in a parallel study to generate consensus classifications used for validation. Multivariable models were applied to assess associations between pancreatic features and age, body weight, adiposity, fat distribution, and technical variables. Mean pancreatic thickness was 10.8 mm (SD 3.1) and was positively associated with body weight. Pancreatic hyperechogenicity was uncommon (8.3%). Quantitative pancreatic intensity was associated with age. Heterogeneous echotexture was common (43%) and, on subjective assessment, was associated with older age and lower transducer frequency. Quantitative texture features were associated with age, pancreatic thickness, pancreas orientation, and transducer frequency. These findings suggest that pancreatic heterogeneity is frequent and age‐associated, and that both subjective and quantitative assessments are influenced by technical factors.

**Abbreviations**
BCS: body condition score
CP: chronic pancreatitis
SAT: subcutaneous adipose thickness
TAT: total abdominal adipose thickness
VAT: visceral adipose thickness

## 1 Introduction

Abdominal ultrasonography is commonly used to assess the canine pancreas, particularly in dogs presenting with gastrointestinal signs. Despite its widespread use, the interpretation of pancreatic ultrasonographic appearance remains challenging. Echogenicity and echotexture are routinely described in clinical reports, yet their biological significance is often unclear, and substantial overlap exists between findings reported in clinically normal dogs and those described in pancreatic disease [1, 2, 3].

Previous studies have demonstrated that pancreatic thickness increases with body weight, whereas pancreatic echogenicity and echotexture show considerable variability in dogs without clinical or clinicopathological evidence of pancreatitis [1, 2]. Relative to peripancreatic fat, most healthy dogs have an isoechoic pancreas (93%), with a small proportion reported as hyperechoic (7%), and no association between echogenicity, age, weight, or body condition score (BCS) [2]. In contrast, dogs with hyperadrenocorticism are more likely to have a hyperechoic pancreas, suggesting that systemic or endocrine factors may influence pancreatic appearance.

Notably, heterogeneous pancreatic echotexture has been reported in up to approximately 40% of clinically normal dogs and is typically characterized by scattered to coalescing hyperechoic foci [1, 2]. Similar echotextural changes have also been described in association with chronic pancreatitis (CP), exocrine pancreatic insufficiency, fibrosis, fat infiltration, and dystrophic mineralization [1, 2, 4, 5]. Given reported post‐mortem prevalences of CP of 25%–34% in dogs, the frequent identification of pancreatic heterogeneity on ultrasound presents a diagnostic challenge [6]. However, limited study reproducibility and the subjective nature of echotexture assessment have hindered more specific clinical interpretation.

The subjective nature of ultrasonographic descriptors remains a major limitation in pancreatic ultrasound interpretation. In human medicine, only moderate interobserver agreement has been reported for pancreatic echogenicity and echotexture assessment, and similar limitations in veterinary radiology remain incompletely characterized [7]. Quantitative image analysis offers a means of objectively characterizing pancreatic echogenicity and echotexture and reducing reliance on subjective assessment. Texture‐based metrics quantify spatial variation in pixel intensity and have been increasingly applied in medical imaging to improve objectivity and reproducibility [8, 9, 10]. However, the application of quantitative ultrasound analysis to the canine pancreas remains limited, and the relative contributions of biological factors, such as age and body composition, versus technical factors have not been clearly established.

Accordingly, the aims of this study were to characterize subjective and quantitative ultrasonographic features of the pancreas in a tertiary‐referral cohort of dogs, to evaluate their associations with age, body weight, adiposity, abdominal fat distribution, and selected technical parameters, and to validate quantitative metrics against subjective classifications.

## 2 Methods

### 2.1 Study Population

This prospective observational study included client‐owned dogs that underwent abdominal ultrasonography at a tertiary referral hospital between January 2016 and January 2019. Inclusion required an ultrasonographic visualization of the pancreas. Dogs were included if the pancreas was adequately visualized. Exclusion criteria were ultrasonographic evidence of acute pancreatitis or pancreatic mass lesions, poor image quality, or clinical instability precluding image acquisition. Body weight and BCS (9‐point scale) were recorded at the time of examination [11]. A documented diagnosis of hyperadrenocorticism was recorded as a potential confounder. Diagnosis of HAC was made by the attending internist at the referral facility based on a combination of clinical signs, routine laboratory testing, and dynamic endocrine function testing.

Ethical approval was obtained from the institutional animal ethics committee (University of Melbourne Faculty of Veterinary and Agricultural Sciences Animal Ethics Committee ID: 1613988).

### 2.2 Ultrasound Image Acquisition

**FIGURE 1:**
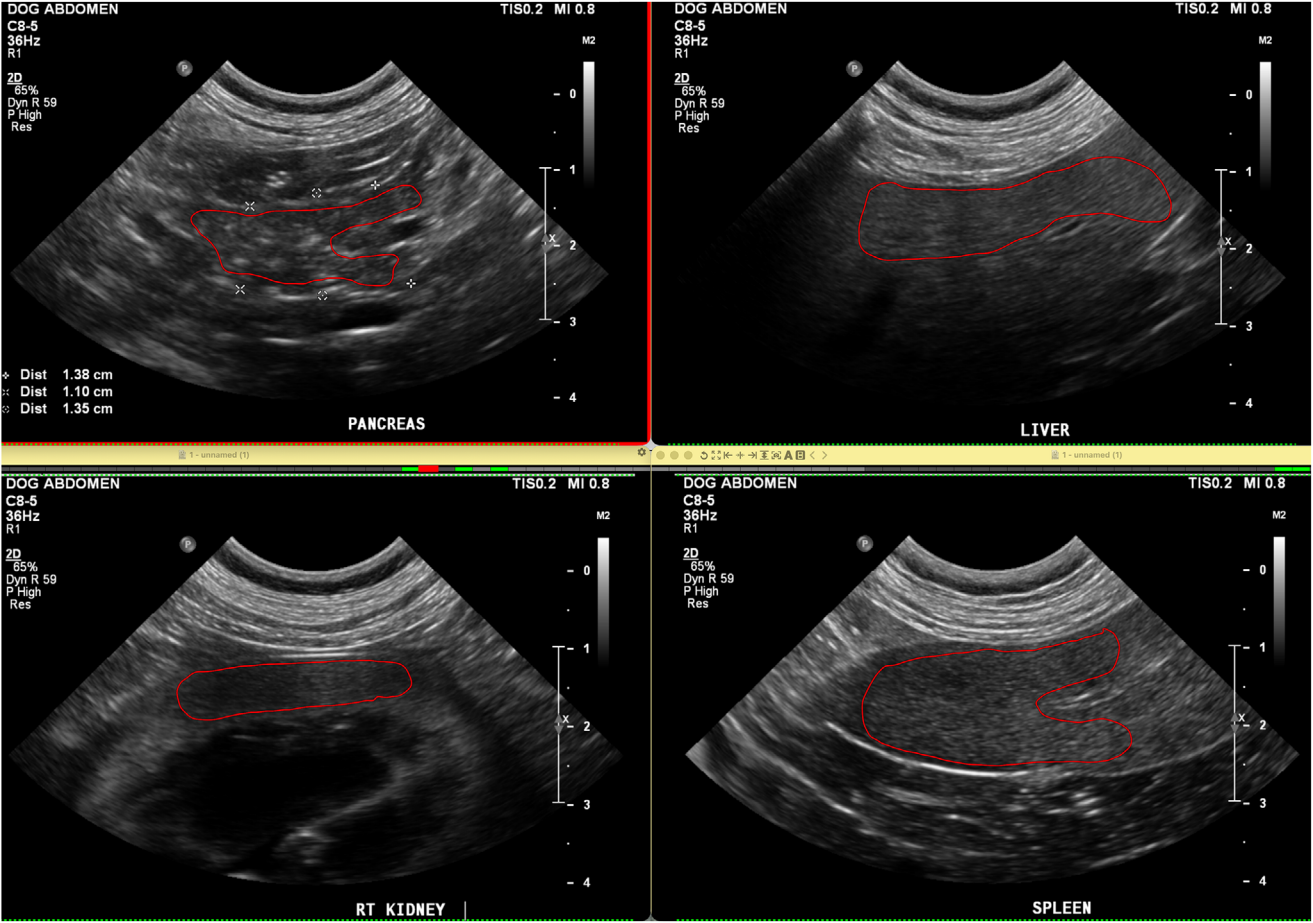
B‐mode examples of regions of interest (ROI) placement in the pancreas, liver, right renal cortex, and spleen for echogenicity for texture analyses.

All ultrasound examinations were performed by a single radiology resident using a standardized protocol on the same ultrasound platform (Philips EPIQ 5). Curvilinear (5–8 MHz) and linear (5–18 MHz) transducers were used according to patient size and acoustic access. Dogs were scanned in dorsal or lateral recumbency using subcostal or intercostal approaches to mitigate gastrointestinal gas and conformation‐related limitations [2, 12]. Images were optimized for either the right lobe or body of the pancreas, and both transverse and longitudinal planes were obtained, when possible, with longitudinal images oriented along the pancreatic duct, if visible [1]. Adjacent liver, right kidney, and spleen images were acquired without altering machine settings, as reference organs (Figure 1). Subjective pancreatic features recorded included size, echogenicity (hypoechoic, isoechoic, hyperechoic), and echotexture (homogeneous, heterogeneous).

### 2.3 Texture Analysis

Quantitative analysis was performed on stored B‐mode images using LIFEx software (version 25.06.01, LITO Institut Curie, Orsay, France) following published radiomics and ultrasound texture analysis recommendations [10, 13]. Freehand regions of interest (ROI) were drawn around representative pancreatic parenchyma, excluding pancreatic ducts, vessels, peripancreatic fat, and artifacts (Figure 1). Reference ROIs were placed in the liver, spleen, and right renal cortex using similar exclusion criteria. Pancreatic ROIs were discretized to 10 gray levels (*b* = 10) following rescaling to 0–255 intensity values, with gray‐level co‐occurrence matrices computed at an offset distance of 1 (*d* = 1) and direction‐averaged for all cases. For sensitivity analysis, a subset of 48 cases was compared with repeated extraction at *b* = 10/*d* = 10 and *b* = 5/*d* = 5. For echogenicity, pancreatic intensity was analyzed as absolute values and as ratios to each reference organ. Second‐order texture features, which are minimally sensitive to absolute intensity scaling, were not normalized [14]. Features were then screened for discrimination, redundancy, and stability before inclusion in statistical models, as detailed in the following section.

### 2.4 Adiposity and Abdominal Fat Distribution

Ultrasound measurements of abdominal adiposity were obtained in a subset of dogs, as previously described [15, 16]. Total abdominal (TAT), visceral (VAT), and subcutaneous (SAT) adipose thickness were measured along the linea alba, 2–4 cm cranial to the umbilicus (TAT = VAT + SAT). Measurements were repeated three times, and the mean was used to calculate the visceral‐to‐fat ratio (VAT/SAT).

### 2.5 Statistical Analysis

A priori sample size calculations (two‐sided *α* = 0.05, 80% power) indicated that the detection of weak‐to‐moderate correlations (*r* = 0.20–0.40) would require approximately 30–194 cases. A planning target of ∼100 cases was therefore adopted, consistent with prior imaging studies [2]. The final sample (*n* = 72) was adequately powered for moderate associations but likely underpowered for small effects.

Statistical analyses were performed in Jamovi (version 2.6.23.0, The Jamovi Project, Sydney, Australia, https://www.jamovi.org) on a single image from each dog. Primary inference was based on complete‐case datasets, and imputed data were used exclusively for sensitivity analyses. Missing values for VAT and SAT fat thickness were imputed jointly using random‐forest imputation (seed = 123; 10 iterations; 100 trees per forest; predictive mean matching with 5 donors), with TAT and VAT/SAT computed passively from each imputed dataset. Sensitivity analyses were performed using imputed data and additional ultrasound‐derived values of adiposity (TAT, imputed‐TAT) and abdominal fat distribution (VAT, VAT‐imputed and VAT/SAT imputed) (Supporting Information S2 section) [17, 18].

Continuous variables were assessed for normality and summarized using appropriate descriptive statistics. Associations between pancreatic features and patient or technical variables were evaluated using Pearson's and Spearman's correlation and multivariable regression (linear, logistic, or ordinal according to outcome type). Variables considered in multivariable models included sex, neuter status, age, body weight, adiposity (BCS), abdominal fat distribution (VAT/SAT), hyperadrenocorticism, pancreatic thickness, image orientation, and transducer frequency. Effect sizes with 95% confidence intervals are reported, and two‐sided *p* < 0.05 was considered statistically significant. Methods of interpretation are summarized in Supporting Information S1 section.

Quantitative features were screened against subjective echotexture (homogeneous vs. heterogeneous). Texture variables that distinguished homogeneous versus heterogeneous echotexture underwent discrimination, redundancy, and stability analyses to reduce multicollinearity and preserve interpretability of the principal component analysis (PCA). Discrimination of heterogeneous versus homogeneous echotexture was assessed using the Mann–Whitney *U* test with rank‐biserial effect size, and receiver operator curve (ROC) and area under the curve (AUC) (95% CI). False discovery rate was controlled at 5% using the Benjamini–Hochberg (BH) procedure (FDR‐adjusted *p* values or *q* values). Texture variables with Pearson's correlations exceeding |0.80| were deemed redundant, and a single representative variable was used for analysis. Sensitivity analysis selected final variables if comparable discretization (bins 10/*d*1, bins 10/*d*10, and bins 5/*d*5) had differences in AUC (|ΔAUC|) less than 0.05 and a non‐significant paired DeLong test [19]. To assess joint effects across multiple quantitative texture outcomes, a multivariate general linear model (MANOVA) was fitted with selected stable texture features entered as a multivariate outcome set. Predictors were prespecified on the basis of biological plausibility and prior subjective echotexture analyses. Omnibus significance was evaluated using Wilks’ Lambda (*α* = 0.05), with Pillai's trace reported as a robustness check. As a sensitivity analysis to address dimensionality and collinearity among texture features, PCA was performed on the four standardized texture variables. Retained component scores were subsequently modeled using the same covariates as the primary MANOVA to assess the robustness of observed associations.

## 3 Results

Results are presented sequentially from subjective assessments to quantitative analyses, followed by multivariate and dimensionality‐reduced modeling of texture features.

### 3.1 Study Population

From January 2016 to January 2019, 540 dog abdominal ultrasounds were performed. After exclusion criteria and incomplete data collection, ultrasounds from 72 dogs were analyzed. The median age, weight, and BCS of the dogs in the study population were 10.0 years (IQR = 4.6 years; range: 0.1–15.8 years); 9.8 kg (IQR = 12.6 kg; range: 2.5–42.0 kg), and 5.0 (IQR = 2.0; range: 2–9), respectively. There were 40 spayed females, 25 neutered males, 6 entire females, and 1 entire male. Five dogs (7%) had a diagnosis of hyperadrenocorticism. Fifteen (21%) VAT and SAT measurements were missing from the dataset. These values were imputed for use in sensitivity analyses, whereas complete‐case data were used for primary inference.

### 3.2 Pancreas Thickness

Mean pancreatic thickness was 10.8 mm (SD = 3.1; range 5.5–19.9 mm). In multivariable analysis, pancreatic thickness increased with body weight (*B* = 0.148 mm/kg, [95% CI: 0.049, 0.246], *p* = 0.003). No important associations were identified between pancreas thickness and the other potentially explanatory variables considered (age, BCS, or abdominal fat distribution, diagnosis of HAC, pancreas orientation, or transducer frequency). See Supporting Information S2 section for plots and sensitivity analyses.

### 3.3 Relationship Between Subjective Echogenicity and Echotexture

The pancreatic echogenicity was hypoechoic in 55 (76%), isoechoic in 11 (15%), and hyperechoic in 6 (8%) of cases. Echotexture was homogeneous in 41 (57%) and heterogeneous in 31 (43%) with comparable distribution within echogenicity categories, although interpretation was limited by a small number of hyperechoic and isoechoic pancreases (Fisher–Freeman–Halton exact, *p* = 0.293, Cramér's *V* = 0.186). Results are summarized in Table 1 and Supporting Information S3 section.

**TABLE 1:** Contingency table of subjective ultrasonographic echogenicity and echotexture classifications in 72 dog pancreases.

	Echotexture		
Echogenicity	Homogeneous N (proportion)	Heterogeneous N (proportion)	Total N (proportion)
Hypoechoic	31 (0.76)	24 (0.77)	55 (0.76)
Isoechoic	8 (0.20)	3 (0.10)	11 (0.15)
Hyperechoic	2 (0.04)	4 (0.13)	6 (0.08)
**Total**	**41 (0.57)**	**31 (0.43)**	**72**

### 3.4 Relationship Between Subjective Echogenicity, Quantitative Echogenicity, and Covariates

**FIGURE 2:**
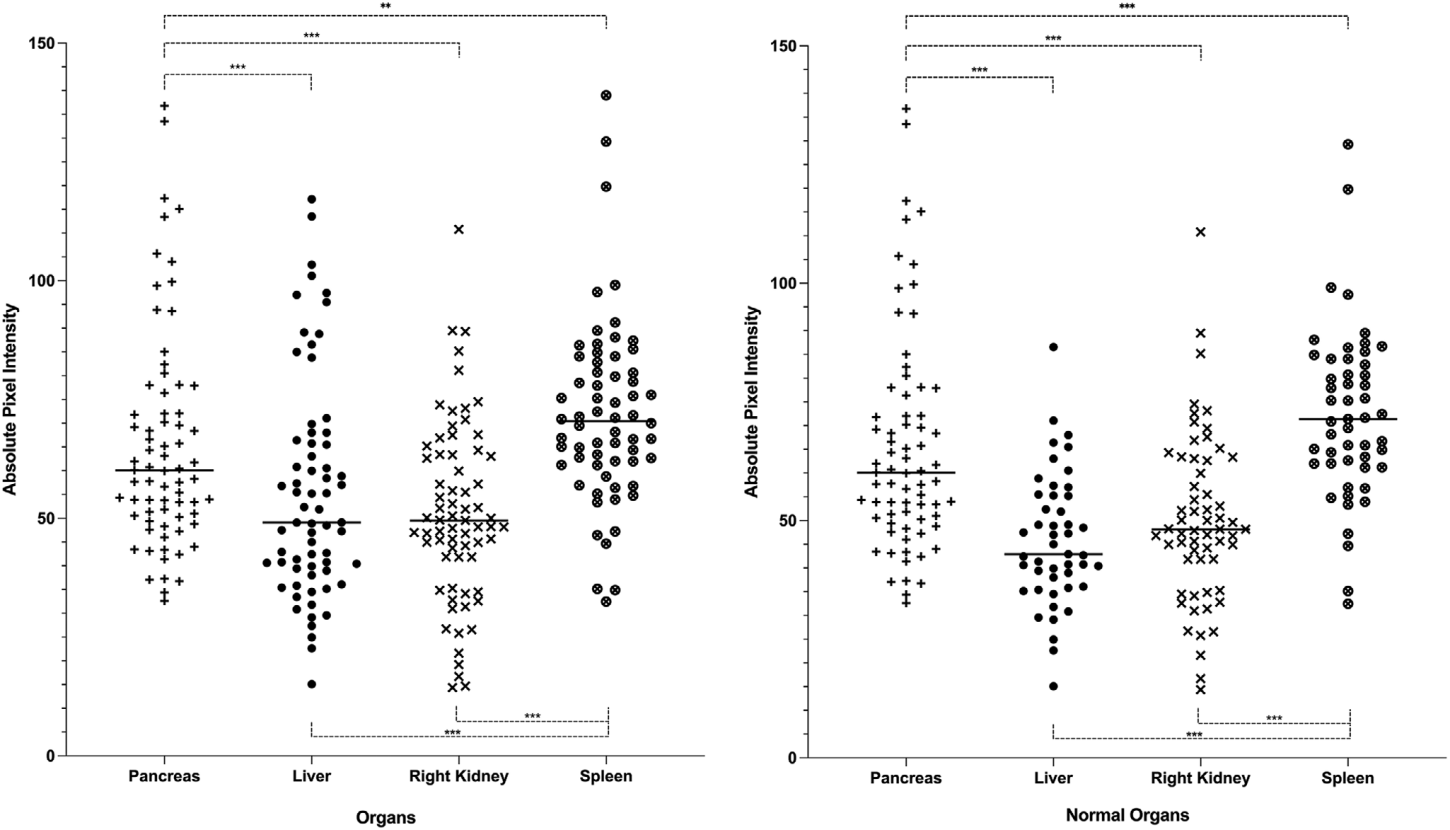
Scatter plots of absolute pixel intensity of paired organs imaged under identical ultrasound settings, with all cases and a subset of cases considered sonographically normal. Pairwise differences were tested with the Wilcoxon signed‐rank test. (Asterisks indicate significance: *p* < 0.05*, *p* < 0.01**, *p* < 0.001***).

Quantitatively assessed pancreatic echogenicity depended on the reference organ. Descriptively, pancreatic absolute pixel intensity (median (mdn) = 60.1) was higher than pixel intensity for the liver (mdn = 49.1; rank‐biserial *r* = 0.65, *p* < 0.001) and right renal cortex (mdn = 49.5; *r* = 0.67, *p* < 0.001), and lower than intensity for the spleen (mdn = 70.5; *r* = −0.45, *p* = 0.002). Across cases, the splenic absolute pixel intensity (IQR = 19.5) and the pancreas‐to‐spleen pixel intensity ratio (entire set IQR = 0.29; sonographically normal subset IQR = 0.28) had the least dispersion, and therefore the pancreas/spleen ratio was used as the primary quantitative metric. Descriptive and sensitivity results are summarized in Supporting Information S2 section and Figure 2.

Increased absolute pixel intensity was associated with higher subjectively‐assessed echogenicity (Kruskal–Wallis *p* < 0.001, *ε* ^2^ = 0.332). Pairwise tests showed hypoechoic pancreases had lower intensity than those pancreases subjectively assessed as isoechoic (*p* = 0.002) and hyperechoic (*p* < 0.001), whereas isoechoic and hyperechoic pancreases were comparable (*p* = 0.167). Ratioed echogenicity mirrored this relationship to subjective echogenicity for pancreas/spleen (*p* < 0.001, *ε* ^2^ = 0.218) and pancreas/right renal cortex (*p* = 0.007, *ε* ^2^ = 0.147), but not for pancreas/liver (*p* = 0.175, *ε* ^2^ = 0.053). Full pairwise and sensitivity results are summarized in Supporting Information S2 section.

In multivariable analysis, subjective echogenicity was not associated with age, weight, adiposity (BCS), fat distribution (VAT/SAT), sex‐neutered status, diagnosis of HAC, pancreas thickness, pancreas orientation, and transducer frequency (all *p* > 0.05) (Table 2). In sensitivity analyses, pancreatic echogenicity was associated with a diagnosis of HAC and inversely related to pancreatic thickness. See Supporting Information S2 section for sensitivity analyses.

**TABLE 2:** Ordinal regression model of subjective pancreatic echogenicity (hypoechoic < isoechoic < hyperechoic) assessing patient characteristics and ultrasound acquisition factors in 72 dogs.

Predictor	OR	OR 95% CI	*p*
Age (years)	1.146	0.927, 1.420	0.21
Weight (kg)	0.995	0.901, 1.100	0.92
Total adiposity (BCS)	1.019	0.78, 1.800	0.95
Visceral adiposity (VAT/SAT)	1.011	0.788, 1.300	0.93
Sex (female vs. male)	1.092	0.208, 5.720	0.92
Neuter status (desexed vs. entire)	2.501	0.206, 30.34	0.47
HAC (Yes vs. No)	10.452	0.630, 173.490	0.10
Pancreas thickness (mm)	0.742	0.541, 1.020	0.06
Pancreas orientation (transverse–longitudinal)	1.454	0.310, 6.820	0.64
Transducer (C8‐5 vs. L18‐5)	1.673	0.293, 9.550	0.56

*Note*: Statistically significant with *p *< 0.05.

Abbreviations: BCS—body condition score; HAC—diagnosed hyperadrenocorticism; OR—odds ratio; SAT—subcutaneous adipose thickness; VAT—visceral adipose thickness.

In multivariable analysis, absolute pancreatic and relative pancreatic/spleen pixel intensity was associated with age (pancreas absolute pixel intensity: *B* = 1.695, [95% CI: 0.216, 3.174], *p* = 0.021; pancreas/spleen relative pixel intensity: *B* = 0.017, [95% CI: 0.000, 0.035], *p* = 0.045), and not associated with weight, adiposity (BCS), fat distribution (VAT/SAT), sex‐neutered status, diagnosis of HAC, pancreas thickness, pancreas orientation, and transducer frequency (all *p* > 0.05). In the sensitivity analysis, VAT was associated with increasing absolute pancreatic and relative pancreatic/spleen pixel intensity. The association between age and relative pancreatic pixel intensity when normalized to other organs (pancreas/liver and pancreas/right kidney) was not replicated in the sensitivity analysis. No other trends were observed in the sensitivity analysis. Results are summarized in Table 3, and the sensitivity analysis in Supporting Information S2 section.

**TABLE 3:** General linear model outcomes for pancreatic absolute pixel intensity and relative intensity to the spleen.

Predictor	Pancreas Absolute Pixel Intensity			Pancreas/Spleen Pixel Intensity Ratio		
	B	95% CI	*p*	B	95% CI	*p*
Age (years)	1.695	0.216, 3.174	0.021^*^	0.017	0.000, 0.034	0.045^*^
Weight (kg)	−0.256	−1.009, 0.497	0.49	0.004	−0.006, 0.013	0.44
Total adiposity (BCS)	−2.125	−6.738, 2.487	0.35	−0.011	−0.065, 0.044	0.70
Visceral adiposity (VAT/SAT)	1.818	−0.430, 4.066	0.10	0.011	−0.015, 0.036	0.41
Sex (Female vs. male)	4.115	−8.504, 16.733	0.51	−0.084	−0.232, 0.064	0.26
Neuter status (desexed vs. entire)	3.076	−18.130, 24.281	0.77	−0.162	−0.399, 0.075	0.18
HAC (Yes vs. No)	6.572	−18.472, 31.615	0.60	0.017	−0.264, 0.298	0.91
Pancreas thickness (mm)	−0.101	−2.134, 1.932	0.92	−0.005	−0.029, 0.019	0.68
Pancreas orientation (Transverse–longitudinal)	0.974	−11.677, 13.624	0.88	0.104	−0.047, 0.256	0.18
Transducer (C8‐5 vs. L18‐5)	9.167	−6.115, 24.449	0.23	0.125	−0.047, 0.297	0.16

*Note: B*—model coefficient.

^*^ Statistically significant with *p*<0.05.

Abbreviations: BCS—Body condition score; HAC—diagnosed hyperadrenocorticism; SAT—subcutaneous adipose thickness; VAT—visceral adipose thickness.

### 3.5 Relationship Between Subjective Echotexture, Quantitative Echotexture, and Covariates

On multivariable logistic regression, the odds of heterogeneous pancreatic echotexture (subjective pancreatic echotexture: homogeneous vs. heterogeneous) increased with age (OR = 1.251, [95% CI: 1.030, 1.519], *p* = 0.024) and decreased with higher transducer frequency (OR = 0.080, [95% CI: 0.007, 0.894], *p* = 0.040), adjusting for other covariates. The sensitivity analysis suggested a positive association with TAT and a negative association with VAT. Results are summarized in Table 4, and the sensitivity analysis in Supporting Information S2 section.

**TABLE 4:** Logistic regression model for associations with subjectively assessed pancreatic echotexture (homogeneous vs. heterogeneous) and patient characteristics and ultrasound acquisition factors in 72 dogs.

Predictor	OR (expB)	95% CI	*p*
Age (per year)	1.251	1.030, 1.519	0.024^a^
Weight (kg)	0.980	0.896, 1.073	0.66
Total adiposity (BCS)	1.091	0.610, 1.951	0.77
Visceral adiposity (VAT/SAT)	0.836	0.627, 1.115	0.22
Sex (female vs. male)	2.542	0.572, 11.302	0.22
Neuter status (desexed vs. entire)	0.604	0.038, 9.543	0.72
HAC (Yes vs. No)	2.633	0.087, 79.690	0.58
Pancreas thickness (mm)	1.109	0.869, 1.416	0.40
Pancreas orientation (transverse–longitudinal)	0.423	0.087, 2.048	0.29
Transducer (C8‐5 vs. L18‐5)	0.080	0.007, 0.894	0.040^a^

Abbreviations: BCS, body condition score; HAC, diagnosed hyperadrenocorticism; OR, odds ratio; SAT, subcutaneous adipose thickness; VAT, visceral adipose thickness.

^a^ Statistically significant with *p* < 0.05.

Quantitative variables that distinguished subjective echotexture (homogeneous vs. heterogeneous), retained after discrimination, redundancy, and sensitivity analyses, included conventional standard deviation, GLRLM_SRE, GLRLM_RLNU, and NGLDM_contrast. A limited number of predictors were selected for multivariate analysis due to low numbers and a priori based on biological plausibility and prior subjective echotexture analyses, which included age, VAT, pancreas thickness, transducer frequency, and pancreas orientation. To limit model complexity relative to sample size, collinear weight (effects choice of transducer) and adiposity measures were excluded. HAC was excluded due to the low number of cases.

Multivariate omnibus testing demonstrated a significant association between transducer frequency and the combined texture feature set (Wilks’ *Λ* = 0.655, *p* < 0.001; Pillai's trace *V* = 0.345, *p* < 0.001). Pancreas thickness was also associated with the multivariate texture outcomes (*Λ* = 0.632, *p* < 0.001; *V* = 0.368, *p* < 0.001). No significant multivariate associations were observed for pancreas orientation, age, or VAT (all *p* > 0.10). Results and sensitivity analyses are summarized in Supporting Information S5 section.

PCA identified a dominant component explaining 49% of total texture variance. In multivariable regression modeling of this component, age (*B* = 0.068, [95% CI: 0.013, 0.123], *p* = 0.016), pancreas thickness (*B* = 0.101, [95% CI: 0.027, 0.176], *p* = 0.008), and pancreas orientation (*B* = −0.622, [95% CI: −1.121, −0.122], *p* = 0.015) were statistically significantly associated with the principal texture dimension, whereas probe frequency and VAT were not. Differences between multivariate and PCA‐based results likely reflect reweighting of texture information in the PCA, which emphasizes the dominant axis of texture variation rather than the full multivariate texture profile. The differing associations observed between logistic regression, MANOVA, and PCA models likely reflect the distinct statistical structures of these approaches. As a result, predictors may be associated with specific quantitative texture dimensions without necessarily influencing subjective classification or the full multivariate texture profile. Results and sensitivity analysis are summarized in Supporting Information S5 section.

## 4 Discussion

In this tertiary cohort, older dogs were more likely to have quantitatively hyperechoic and both subjective and quantitatively heterogeneous pancreases on ultrasound. Pancreatic heterogeneity also increased with thicker pancreases (quantitatively) and lower transducer frequency (subjectively and quantitatively), independent of adiposity measures. Additionally, in PCA, features of heterogeneity were associated with pancreas orientation. Further, quantitative texture metrics reflecting gray‐level variation and spatial organization (e.g., standard deviation, entropy, energy, and several neighborhood‐based statistical texture descriptors) were correlated with subjective assessment of heterogeneity. In combination, quantitative measures complement subjective assessment and may provide measures of objective pancreatic evaluation in clinical practice and research.

The relationships between sonographic pancreatic characteristics and patient factors identified in this study both extend and, in some respects, challenge previous reports. Consistent with the findings of Penninck et al., a positive correlation was observed between body weight and pancreatic thickness, and further, we showed no correlation with pancreatic size, adiposity, and abdominal fat distribution [1]. Further, the pancreatic subjective echogenicity mirrored previous studies, with most of the pancreases hypo‐ to isoechoic to peripancreatic fat (92% of cases), hyperechoic to liver and right renal cortex, and hypoechoic to spleen [20, 21]. Finally, this study reaffirmed the absence of any association between pancreatic echogenicity or echotexture and adiposity (BCS) and showed no relationship with abdominal fat distribution [2].

Although pancreatic health was not specifically defined and the sampled population likely included a broader clinical spectrum, the prevalence of pancreatic hyperechogenicity (8%; 6/72) and heterogeneity (43%; 31/72) closely paralleled those reported by Granger et al. (7% and 40%, respectively) and exceeded the 8% heterogeneity described by Penninck et al. [1, 2]. When dogs with known hyperadrenocorticism were excluded, the prevalence of hyperechogenicity decreased to 4% (3/69), whereas heterogeneity remained similar (42%; 29/69), mirroring the inclusion criteria of the first part of Granger's study [2]. Although the clinical relevance and underlying causes of pancreatic hyperechogenicity remain uncertain, its relative rarity suggests that potential associations should be carefully considered before the finding is dismissed, particularly with hypercortisolaemia [2, 22]. In this study, dogs with hyperadrenocorticism had higher estimated odds of pancreatic hyperechogenicity (OR = 10.45); however, this finding was based on only five affected dogs and was accompanied by a very wide confidence interval (95% CI: 0.63–173.49), precluding definitive inference. The similar direction and magnitude of effect reported by Granger et al. (OR = 9.28) suggest a possible corticosteroid‐associated influence on pancreatic echogenicity, but confirmation in adequately powered studies is required.

The relationship between pancreatic heterogeneity and CP remains complex, particularly given the high prevalence of heterogeneity in clinically normal dogs (40%–42%) [2]. Reported prevalences in dogs with pancreatitis or histologically confirmed CP range from approximately 25–57%, but many studies lack control groups, consistent diagnostic criteria, or histopathological correlation, limiting interpretation [5, 6, 23, 24]. Although heterogeneity may reflect fibrosis, inflammation, or mineralization, inconsistent definitions and the absence of standardized criteria, such as those used in human medicine, likely contribute to variable findings [7, 25]. Without histological validation, heterogeneous echotexture may represent CP, age‐related change, corticosteroid effects, fatty infiltration, or normal anatomical variation [26, 27].

Quantitative measures of increased heterogeneity were associated with increasing pancreatic thickness. This was unexpected and was not appreciated subjectively. Although this finding may be spurious, several explanations are possible. Theoretically, a thicker pancreas may result in a larger insonated cross‐sectional area, increasing the number of lobules, ducts, and septal interfaces sampled within a single imaging plane, which may amplify gray‐level variation independent of overt pathology [20, 28]. Increased depth may also alter attenuation and speckle characteristics, contributing to apparent heterogeneity. Although interlobular fibrosis or connective tissue expansion could theoretically increase echotextural variation, CP is typically associated with pancreatic atrophy rather than enlargement, making it a less likely cause of the heterogeneity [3, 29]. The absence of a subjective association further suggests that this may represent sub‐visual quantitative variation or a methodological effect. Replication in larger cohorts will be required to determine whether this finding reflects true biological variation or an imaging artifact.

Regarding the relationship between age and pancreatic echogenicity and echotexture, the present study both reinforces and diverges from previous findings. Granger et al. reported no association between age and either echogenicity or echotexture [2]. Similarly, the current study found no significant relationship between subjective echogenicity, but absolute and relative (pancreas/spleen pixel intensity ratio) intensities were positively associated with age. This association was not reproduced when the pancreas was ratioed to the liver or right renal cortex, suggesting that any apparent effect of age on echogenicity is reference‐dependent. Importantly, these reference organs themselves may undergo age‐ or disease‐related echogenic change, such as vacuolar hepatopathy or chronic kidney disease, or be influenced by anisotropy artifact, potentially confounding ratio‐based comparison [20]. If an age‐related increase in pancreatic echogenicity does exist, as consistently demonstrated in human populations, it will require confirmation in larger dog cohorts [27].

Pancreatic echotexture showed a positive association with age in this study, differing from one dog study but consistent with human findings, where parenchymal heterogeneity increases with advancing age [2, 27]. The underlying microstructural cause was not determined but may reflect the rising histological prevalence of CP with age [6]. This suggests that age‐related heterogeneity could represent a continuum, from subclinical or chronic idiopathic (“senile”) pancreatitis to clinically significant CP, or may simply reflect benign age‐associated parenchymal remodeling [6, 27]. Longitudinal and histopathologically correlated studies are warranted to clarify whether these sonographic changes indicate disease or normal aging.

For ultrasound to be used effectively in assessing the pancreas, the interaction between acoustic parameters and pancreatic microstructure must be understood. In this study, transducer frequency and orientation had no effect on pancreatic echogenicity. However, it is feasible that higher frequency and transducer orientation may affect echogenicity, through increasing backscatter from Rayleigh scattering, and anisotropic effects with radiating arrangement of the fibrovascular capsule, interlobular septa, ducts, or acini [30, 31]. For this reason, transducer frequency and orientation should remain a consideration during pancreatic evaluation and warrant further investigation.

In contrast, the relationship between transducer frequency and echotexture was consistent, with pancreases more likely to appear homogeneous at higher transducer frequencies. This likely reflects improved spatial resolution, increased backscatter/specular reflection, and less beam distortion or side‐lobe artifact at higher frequencies [20]. In addition, finer scattering structures may become individually resolvable, giving the impression of greater textural uniformity [20]. These results indicate that, where possible, the pancreas should be examined at the highest feasible frequency, and that greater diagnostic weight may be placed on subjective heterogeneity in such settings. The influence of imaging parameters on both subjective and quantitative textural features is well recognized in the human and veterinary radiomics literature [32, 33]. Further research should determine which quantitative image metrics remain robust across ultrasound settings and platforms to enable standardized, reproducible assessment of pancreatic echotexture [33].

Regarding texture analysis, several first‐ and second‐order features proved discriminative and sensitive for detecting pancreatic heterogeneity, consistent with previous radiomic studies [34, 35]. In addition, some texture variables correlated with patient factors that were not evident on subjective assessment, suggesting that quantitative analysis may capture microstructural differences beyond visual perception [35]. Aside from relatively consistent associations of radiomic features with transducer frequency and age, the significance of other correlations remains uncertain in the absence of histologic or clinical reference standards and may represent spurious or confounded effects. Nonetheless, these findings highlight technical and biological factors that should be considered in future research aimed at standardizing and validating quantitative pancreatic ultrasound.

This study has several limitations. The population was drawn from a tertiary referral center; histopathological confirmation was not available, adiposity data were incomplete in some dogs, and residual confounding cannot be excluded. Although image acquisition was standardized on a single ultrasound platform, radiomic values may differ across systems and signal‐processing settings [36]. Prospective validation in independent cohorts, ideally incorporating standardized reporting templates, radiomic guidelines, clinical outcomes, and histologic reference standards, is needed before quantitative metrics can be confidently applied in clinical practice [37, 38].

## 5 Conclusions

Pancreatic hyperechogenicity was uncommon, whereas heterogeneity was frequently observed. Increasing age was associated with both quantitative echogenicity and heterogeneity, and lower transducer frequency was associated with subjective heterogeneity. Quantitative texture variation was also associated with pancreatic thickness. Adiposity and abdominal fat distribution were not associated with pancreatic echogenicity or echotexture. These findings highlight the influence of age and technical factors on pancreatic ultrasound appearance and support the need for standardized acquisition and external validation before quantitative biomarkers can distinguish normal variation from clinically significant pancreatic disease.

## Author Contributions

Conception and design: Robert B. S. Turner. Acquisition of data: Robert B. S. Turner. Analysis and interpretation of data: Robert B. S. Turner and Simon M. Firestone. Drafting the article: Robert B. S. Turner. Revising article for intellectual content: Robert B. S. Turner, Simon M. Firestone, Caroline Mansfield, and Frank R. Dunshea. Final approval of the completed article: Robert B. S. Turner, Simon M. Firestone, Caroline Mansfield, and Frank R. Dunshea.

## Disclosure

This manuscript forms part of a doctoral thesis to be submitted to the University of Melbourne.

## Ethics Statement

Ethical approval was obtained from the University of Melbourne Faculty of Veterinary and Agricultural Sciences Animal Ethics Committee ID: 1613988.

## Conflicts of Interest

The authors declare no conflicts of interest.

## Supporting information




**Supplementary File 1**: vru70208‐Supp‐0001‐SuppMat1.docx

**Supplementary File 2**: vru70208‐Supp‐0002‐SuppMat2.docx

**Supplementary File 3**: vru70208‐Supp‐0003‐SuppMat3.docx

**Supplementary File 4**: vru70208‐Supp‐0004‐SuppMat4.docx

**Supplementary File 5**: vru70208‐Supp‐0005‐SuppMat5.docx

**Supplementary File 6**: vru70208‐Supp‐0005‐SuppMat6.docx

## Data Availability

The data that support the findings of this study are available from the corresponding author upon reasonable request.
