# Supplementary material for: Effect of Body Composition and Age on the Subjective and Quantitative Ultrasound Appearance of the Dogs’ Pancreas
Source: Vet Radiol Ultrasound. 2026 Jul 15;67(4):e70208. doi: 10.1111/vru.70208 (PMC13371151; doi:10.1111/vru.70208)
Supplement: Supplementary file 1 — vru70208‐Supp‐0001‐SuppMat1.docx [file VRU-67-0-s001.docx]

S1. Interpretation Categories

**Spearman (ρ) and Pearson (*r*) correlations**

Interpretation based on Schober’s summarised guidelines (1). Interpretation applies to |r| or |ρ| (absolute value) and is context-dependent.

| **Correlation** | **Spearman (ρ) and Pearson (*r*) Coefficient Value** |
| --- | --- |
| Negligible | 0.00–0.10 |
| Weak | 0.10–0.39 |
| Moderate | 0.40–0.69 |
| Strong | 0.70–0.89 |
| Very strong | 0.90–1.00 |

1. Schober P, Boer C, Schwarte LA. Correlation Coefficients: Appropriate Use and Interpretation. Anesthesia and analgesia. 2018;126(5):1763-8.
