# Supplementary material for: Effect of Body Composition and Age on the Subjective and Quantitative Ultrasound Appearance of the Dogs’ Pancreas
Source: Vet Radiol Ultrasound. 2026 Jul 15;67(4):e70208. doi: 10.1111/vru.70208 (PMC13371151; doi:10.1111/vru.70208)
Supplement: Supplementary file 2 — vru70208‐Supp‐0002‐SuppMat2.docx [file VRU-67-0-s006.docx]

S2. Summary of Descriptive and Inferential Statistics with Sensitivity Analysis for Quantitative Ultrasound Measures of the Canine Pancreas (Thickness, Echogenicity, Echotexture)

**Key**

*B*  Unstandardised Coefficient

BCS Body condition score

DSCF Dwass-Steel-Critchlow-Fligner pairwise comparisons

FDR False Discovery Rate

HAC Hyperadrenocorticism

IQR Interquartile range

OR Odds ratio

ROI Region of interest

SAT Subcutaneous adipose thickness

VAT Visceral adipose thickness

1. **Pancreatic Thickness**
2. Graph1: Adjusted Trend of Pancreatic Thickness vs Body Weight (95% CI) for 72 dogs


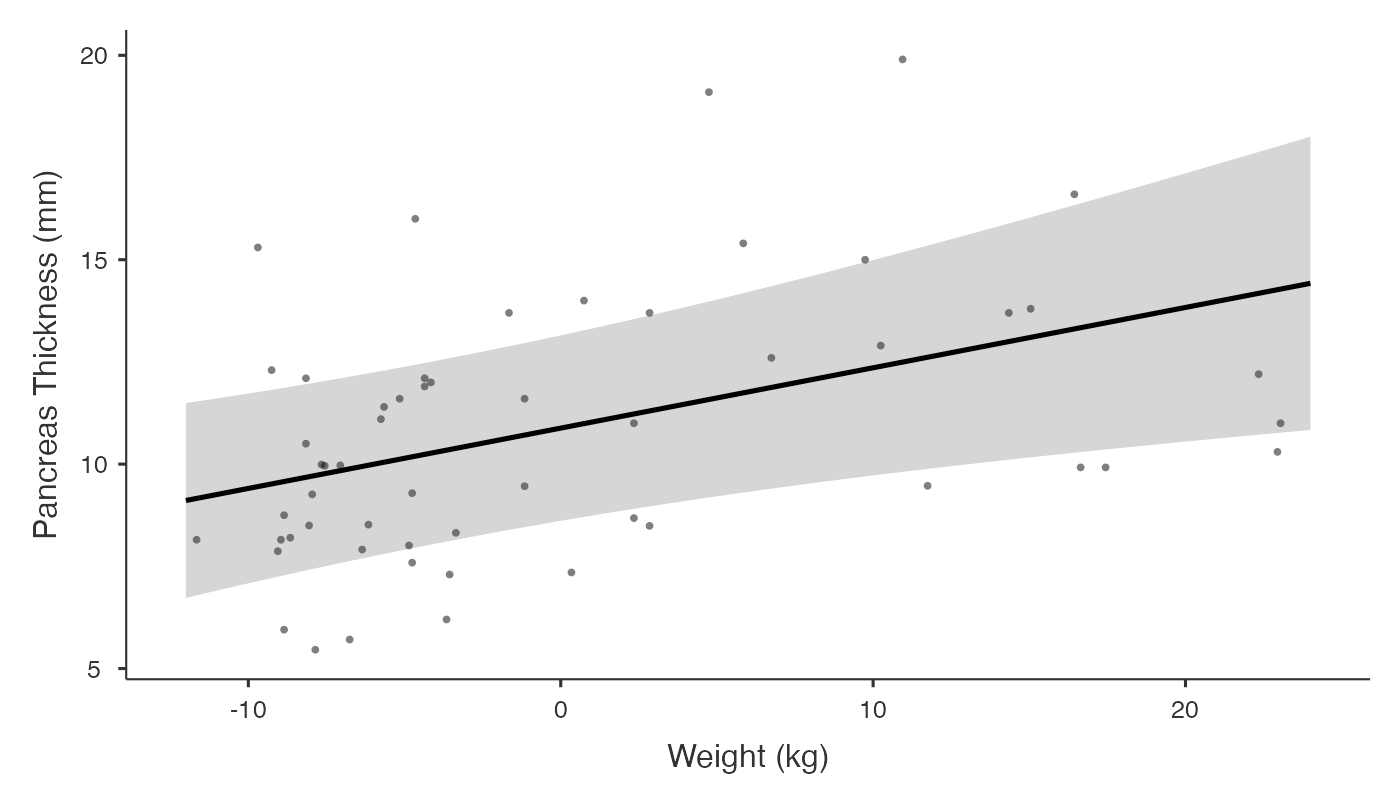


Points are observed values. The line shows model-adjusted predictions with 95% CI from the multivariable linear model (adjusted for age, BCS, VAT/SAT, sex and neuter status, diagnosis of HAC, pancreas orientation, and transducer frequency); Weight *B* = 0.148 mm/kg (95% CI 0.049, 0.246), **p = 0.003**

Table 1: Sensitivity analysis for the body weight–pancreatic thickness association in multivariable models.

| Model Substitutions | Unstandardised Coefficient (*B)* | Unstandardised Coefficient (*B)* 95% CI | p-value |
| --- | --- | --- | --- |
| BCS + complete VAT/SAT  (primary analysis) | 0.148 | 0.049, 0.246 | **0.003*** |
| BCS + imputed VAT/SAT | 0.113 | 0.032, 0.193 | **0.006*** |
| BCS + complete VAT | 0.158 | 0.062, 0.254 | **0.001*** |
| BCS + imputed VAT | 0.140 | 0.058, 0.222 | **<0.001*** |
| Complete TAT + complete VAT | 0.202 | 0.087, 0.316 | **<0.001*** |
| Imputed TAT + imputed VAT | 0.134 | 0.031, 0.237 | **0.011*** |

*statistically significant with p < 0.05

Model: pancreas thickness = intercept + age + weight + substituted adiposity (BCS, complete-TAT, imputed-TAT) + substituted fat distribution (complete-VAT/SAT, imputed-VAT/SAT, complete-VAT, imputed-VAT) + sex + neuter status + HAC diagnosis + image orientation + transducer frequency.

1. Graph 2: Adjusted Trend of Pancreatic Thickness vs Body Condition Score (95% CI)


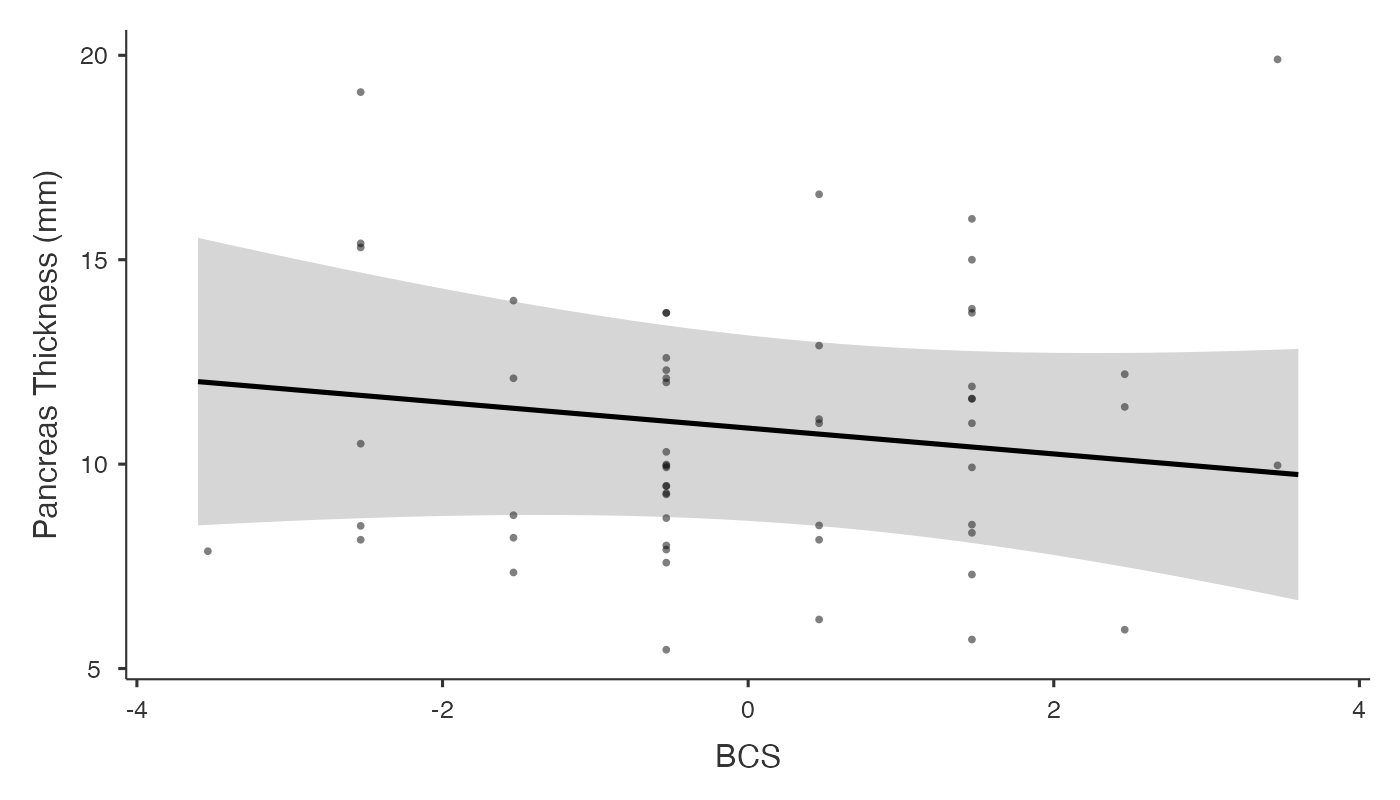


Points are observed values. The line shows model-adjusted predictions with 95% CI from the multivariable linear model (adjusted for age, weight, VAT/SAT, sex and neuter status, diagnosis of HAC, pancreas orientation, and transducer frequency); BCS *B* = -0.316mm/BCS (95% CI -0.965, 0.333), p = 0.340.

Table 2: Sensitivity analysis for the adiposity–pancreatic thickness association in multivariable models.

| Model Substitutions | Unstandardised Coefficient (*B)* | Unstandardised Coefficient (*B)* 95% CI | p-value |
| --- | --- | --- | --- |
| BCS + complete VAT/SAT  (primary analysis) | -0.316 | -0.965, 0.333 | 0.340 |
| BCS + imputed VAT/SAT | -0.273 | -0.869, 0.323 | 0.369 |
| BCS + complete VAT | -0.247 | -0.921., 0.427 | 0.473 |
| BCS + imputed VAT | -0.181 | -0.778, 0.417 | 0.553 |
| Complete TAT + complete VAT | -0.334 | -0.776, 0.108 | 0.139 |
| Imputed TAT + imputed VAT | 0.008 | -0.455, 0.471 | 0.972 |

Model: pancreas thickness = intercept + age + weight + substituted adiposity (BCS, complete-TAT, imputed-TAT) + substituted fat distribution (complete-VAT/SAT, imputed-VAT/SAT, complete-VAT, imputed-VAT) + sex + neuter status + HAC diagnosis + image orientation + transducer frequency.

1. Graph 3: Adjusted Trend of Pancreatic Thickness vs Abdominal Fat Distribution (95% CI)


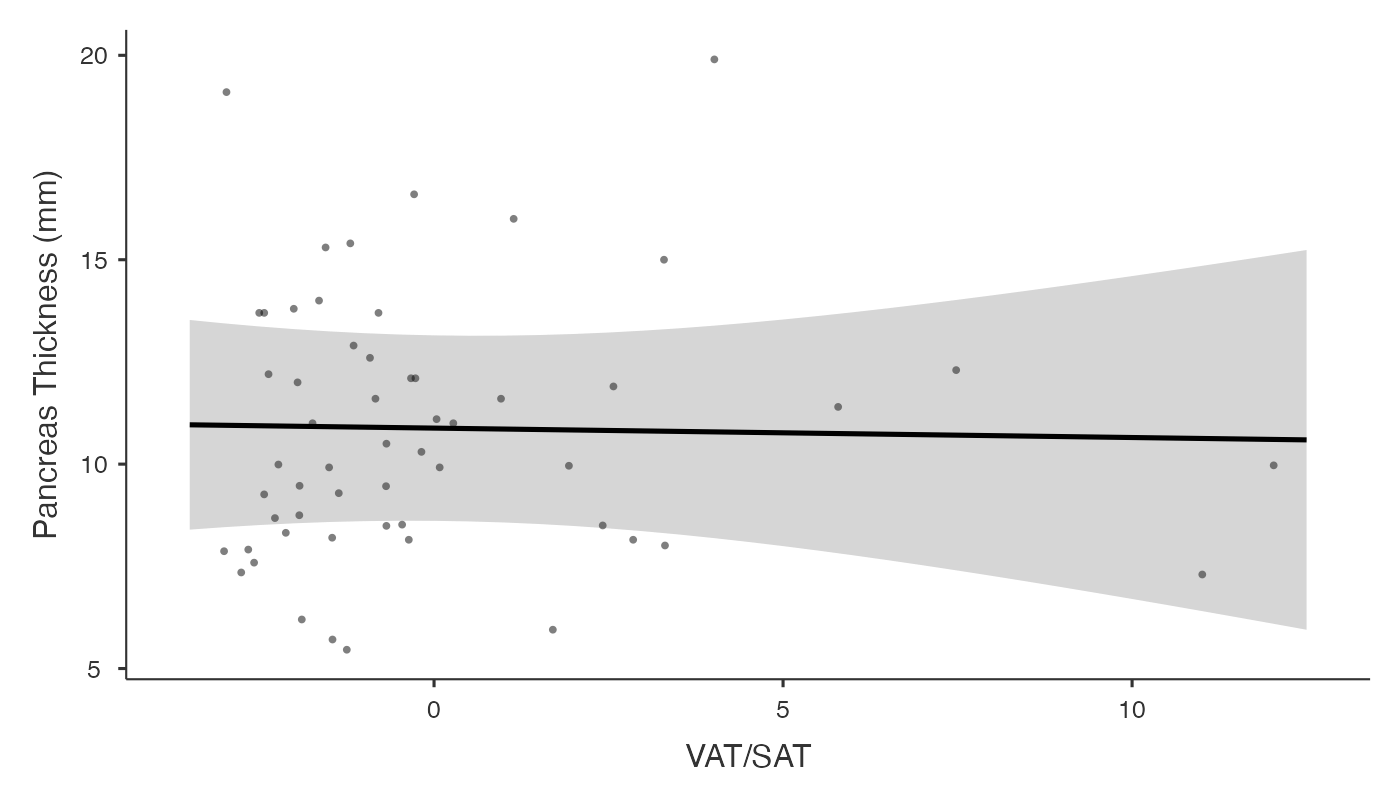


Points are observed values. The line shows model-adjusted predictions with 95% CI from the multivariable linear model (adjusted for age, weight, BCS, sex and neuter status, diagnosis of HAC, pancreas orientation, and transducer frequency); VAT/SAT *B* = -0.023 mm/BCS (95% CI -0.343, 0.296, p = 0.888.

Table 3: Sensitivity analysis for the abdominal fat distribution–pancreatic thickness association in multivariable models.

| Model Substitutions | Unstandardised Coefficient (*B)* | Unstandardised Coefficient (*B)* 95% CI | p-value |
| --- | --- | --- | --- |
| BCS + complete VAT/SAT  (primary analysis) | -0.023 | -0.343, 0.296 | 0.888 |
| BCS + imputed VAT/SAT | -0.148 | -0.411, 0.115 | 0.270 |
| BCS + complete VAT | -0.042 | -0.195, 0.111 | 0.590 |
| BCS + imputed VAT | -0.103 | -0.221, 0.016 | 0.090 |
| Complete TAT + complete VAT | 0.255 | -0.194, 0.703 | 0.266 |
| Imputed TAT + imputed VAT | -0.127 | -0.564, 0.311 | 0.570 |

Model: pancreas thickness = intercept + age + weight + substituted adiposity (BCS, complete-TAT, imputed-TAT) + substituted fat distribution (complete-VAT/SAT, imputed-VAT/SAT, complete-VAT, imputed-VAT) + sex + neuter status + HAC diagnosis + image orientation + transducer frequency.

1. Graph 4: Adjusted Trend of Pancreatic Thickness vs Age (95% CI)


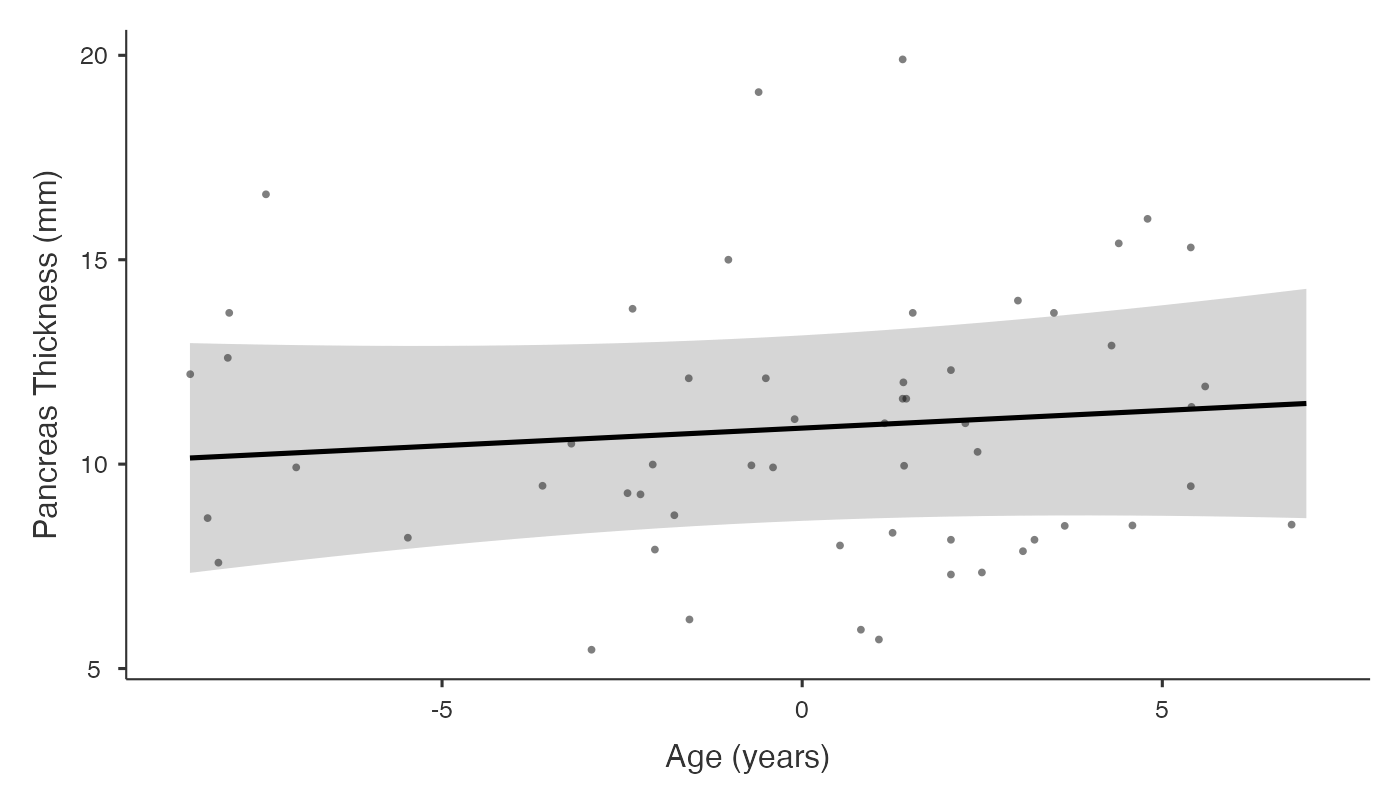


Points are observed values. The line shows model-adjusted predictions with 95% CI from the multivariable linear model (adjusted for weight, BCS, imputed-VAT/SAT, sex and neuter status, diagnosis of HAC, pancreas orientation, and transducer frequency); Age *B* = 0.086mm/yr (95% CI -0.123, 0.295), p = 0.419.

Table 4: Sensitivity analysis for the age–pancreatic thickness association in multivariable models.

| Model Substitutions | Unstandardised Coefficient (*B)* | Unstandardised Coefficient (*B)* 95% CI | p-value |
| --- | --- | --- | --- |
| BCS + complete VAT/SAT  (primary analysis) | 0.086 | -0.123, 0.295 | 0.419 |
| BCS + imputed VAT/SAT | 0.089 | -0.095, 0.272 | 0.346 |
| BCS + complete VAT | 0.086 | -0.116, 0.287 | 0.406 |
| BCS + imputed VAT | 0.102 | -0.081, 0.285 | 0.274 |
| Complete TAT + complete VAT | 0.062 | -0.140, 0.263 | 0.547 |
| Imputed TAT + imputed VAT | 0.107 | -0.076, 0.289 | 0.251 |

Model: pancreas thickness = intercept + age + weight + substituted adiposity (BCS, complete-TAT, imputed-TAT) + substituted fat distribution (complete-VAT/SAT, imputed-VAT/SAT, complete-VAT, imputed-VAT) + sex + neuter status + HAC diagnosis + image orientation + transducer frequency.

1. Graph 5: Adjusted Predictions of Pancreatic Thickness by Sex (95% CI)


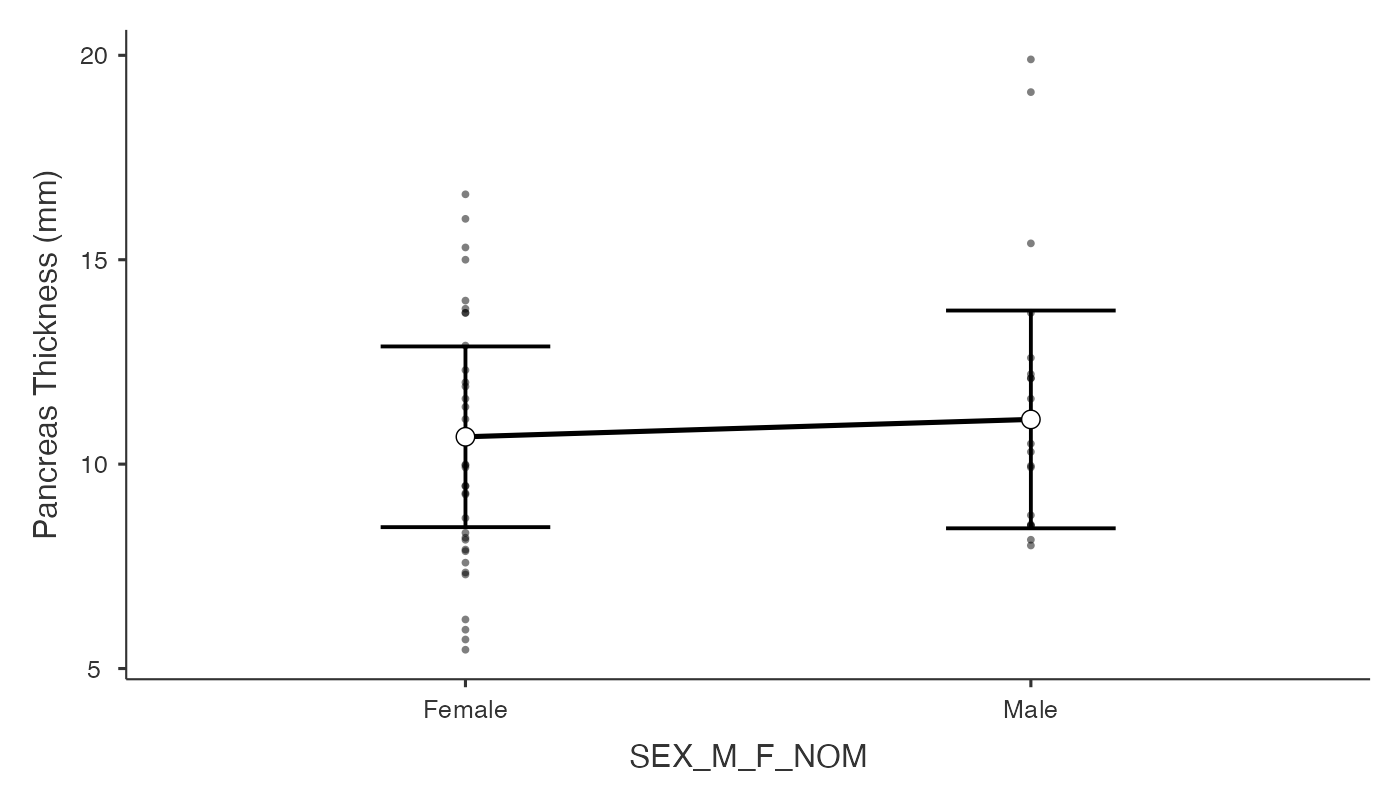


Points are observed values. The line shows model-adjusted predictions with 95% CI from the multivariable linear model (adjusted for age, weight, BCS, VAT/SAT, neuter status, diagnosis of HAC, pancreas orientation, and transducer frequency); Sex status *B* = 0. 425 (95% CI -1.364, 2.214), p = 0.642.

Table 5: Sensitivity analysis for the sex–pancreatic thickness association in multivariable models.

| Model Substitutions | Unstandardised Coefficient (*B)* | Unstandardised Coefficient (*B)* 95% CI | p-value |
| --- | --- | --- | --- |
| BCS + complete VAT/SAT  (primary analysis) | 0.425 | -1.364, 2.214 | 0. 642 |
| BCS + imputed VAT/SAT | 0.215 | -1.293, 1.722 | 0.780 |
| BCS + complete VAT | 0.292 | -1.448, 2.032 | 0.742 |
| BCS + imputed VAT | 0.079 | -1.404, 1.562 | 0.917 |
| Complete TAT + complete VAT | -0.242 | -2.124, 1.639 | 0.801 |
| Imputed TAT + imputed VAT | 0.194 | -1.369, 1.756 | 0.808 |

Model: pancreas thickness = intercept + age + weight + substituted adiposity (BCS, complete-TAT, imputed-TAT) + substituted fat distribution (complete-VAT/SAT, imputed-VAT/SAT, complete-VAT, imputed-VAT) + sex + neuter status + HAC diagnosis + image orientation + transducer frequency.

1. Graph 6: Adjusted Predictions of Pancreatic Thickness by Neuter Status (95% CI)


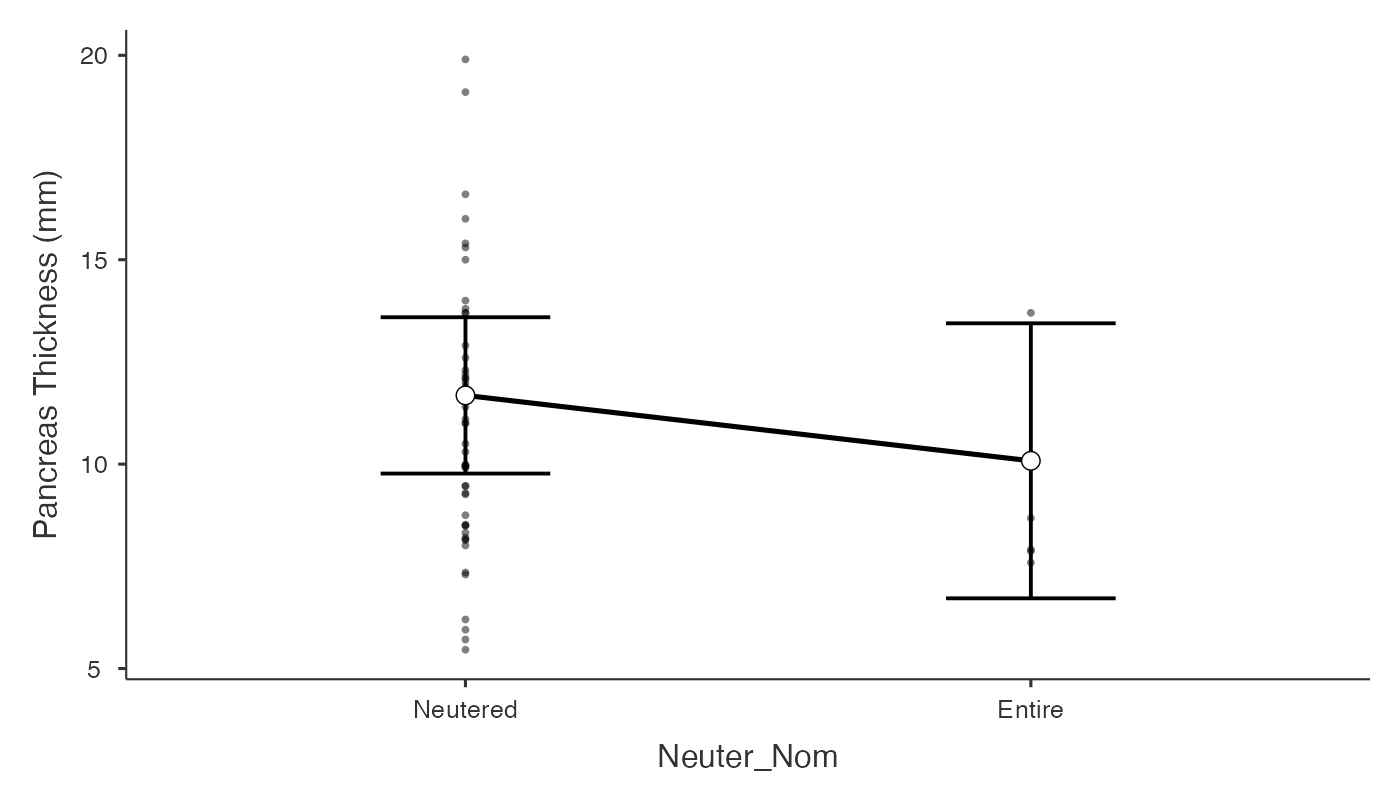


Points are observed values. The line shows model-adjusted predictions with 95% CI from the multivariable linear model (adjusted for age, weight, BCS, VAT/SAT, sex status, diagnosis of HAC, pancreas orientation, and transducer frequency); Neuter status *B* = -1.600 (95% CI -4.579, 1.378), p = 0.292.

Table 6: Sensitivity analysis for the neuter status–pancreatic thickness association in multivariable models.

| Model Substitutions | Unstandardised Coefficient (*B)* | Unstandardised Coefficient (*B)* 95% CI | p-value |
| --- | --- | --- | --- |
| BCS + complete VAT/SAT  (primary analysis) | -1.600 | -4.579, 1.378 | 0. 292 |
| BCS + imputed VAT/SAT | -1.316 | -3742, 1.110 | 0.288 |
| BCS + complete VAT | -1.824 | -4.829, 1.181 | 0.234 |
| BCS + imputed VAT | -1.518 | -4.031, 0.834 | 0.198 |
| Complete TAT + complete VAT | -1.958 | -4.917., 1.002 | 0.195 |
| Imputed TAT + imputed VAT | -1.505 | -3.960, 0.950 | 0.230 |

Model: pancreas thickness = intercept + age + weight + substituted adiposity (BCS, complete-TAT, imputed-TAT) + substituted fat distribution (complete-VAT/SAT, imputed-VAT/SAT, complete-VAT, imputed-VAT) + sex + neuter status + HAC diagnosis + image orientation + transducer frequency.

1. Graph 7: Adjusted Predictions of Pancreatic Thickness by HAC Status (95% CI)


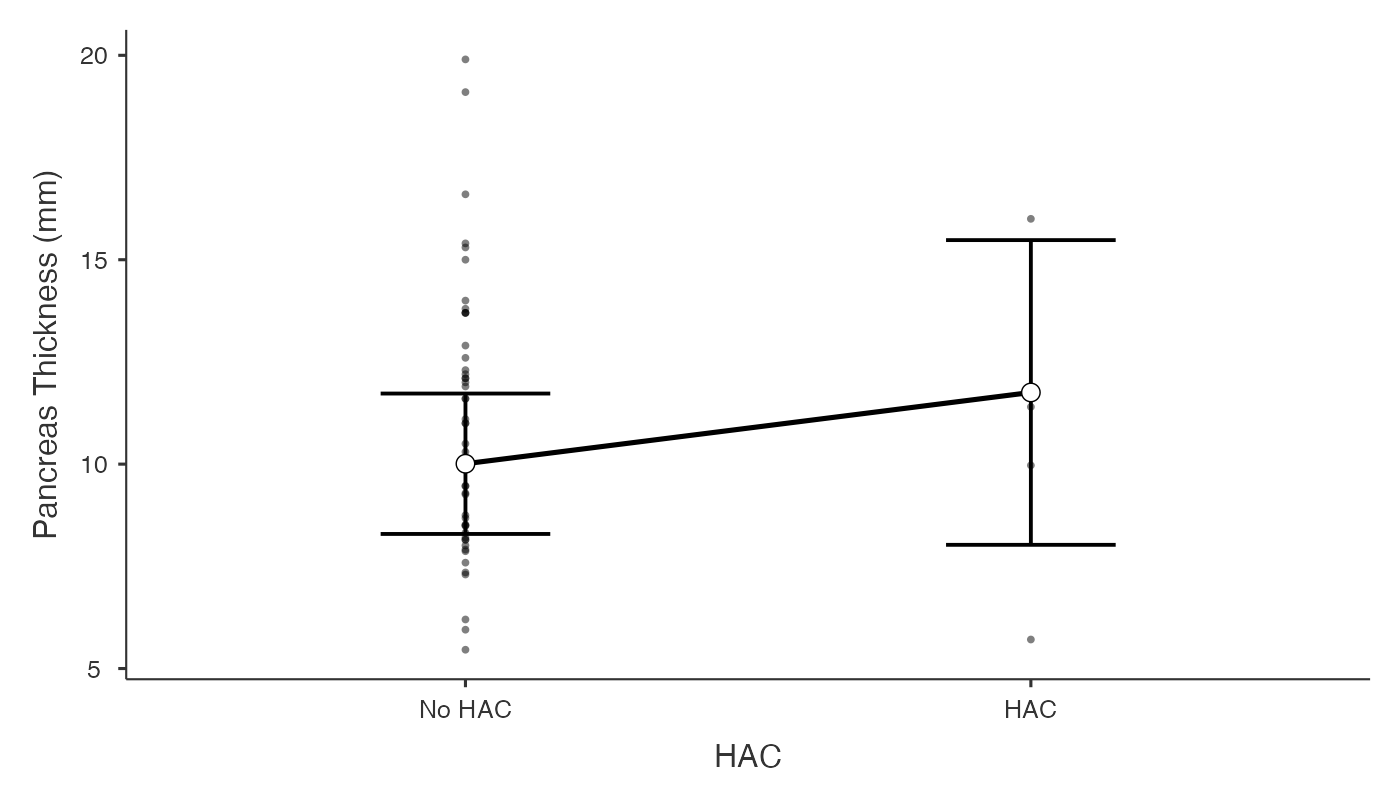


Points are observed values. Markers/line are model-estimated marginal means with 95% CI (adjusted for age, weight, BCS, imputed-VAT/SAT, sex and neuter status, pancreas orientation, transducer frequency); Δ(HAC − No HAC) = *B* = 1.744 mm (95% CI -1.780, 5.267), p = 0.332

Table 7: Sensitivity analysis for the HAC–pancreatic thickness association in multivariable models.

| Model Substitutions | Unstandardised Coefficient (*B)* | Unstandardised Coefficient (*B)* 95% CI | p-value |
| --- | --- | --- | --- |
| BCS + complete VAT/SAT  (primary analysis) | 1.744 | -1.780, 5.267 | 0.332 |
| BCS + imputed VAT/SAT | 1.282 | -1.614, 4.179 | 0.386 |
| BCS + complete VAT | 1.789 | -1.629, 5.207 | 0.305 |
| BCS + imputed VAT | 1.208 | -1.639, 4.054 | 0.406 |
| Complete TAT + complete VAT | 1.183 | -1.998, 4.364 | 0.466 |
| Imputed TAT + imputed VAT | 0.938 | -1.722, 3.649 | 0.498 |

Model: pancreas thickness = intercept + age + weight + substituted adiposity (BCS, complete-TAT, imputed-TAT) + substituted fat distribution (complete-VAT/SAT, imputed-VAT/SAT, complete-VAT, imputed-VAT) + sex + neuter status + HAC diagnosis + image orientation + transducer frequency.

1. Graph 8: Adjusted Predictions of Pancreatic Thickness by Transducers Orientation to the Pancreas (95% CI)


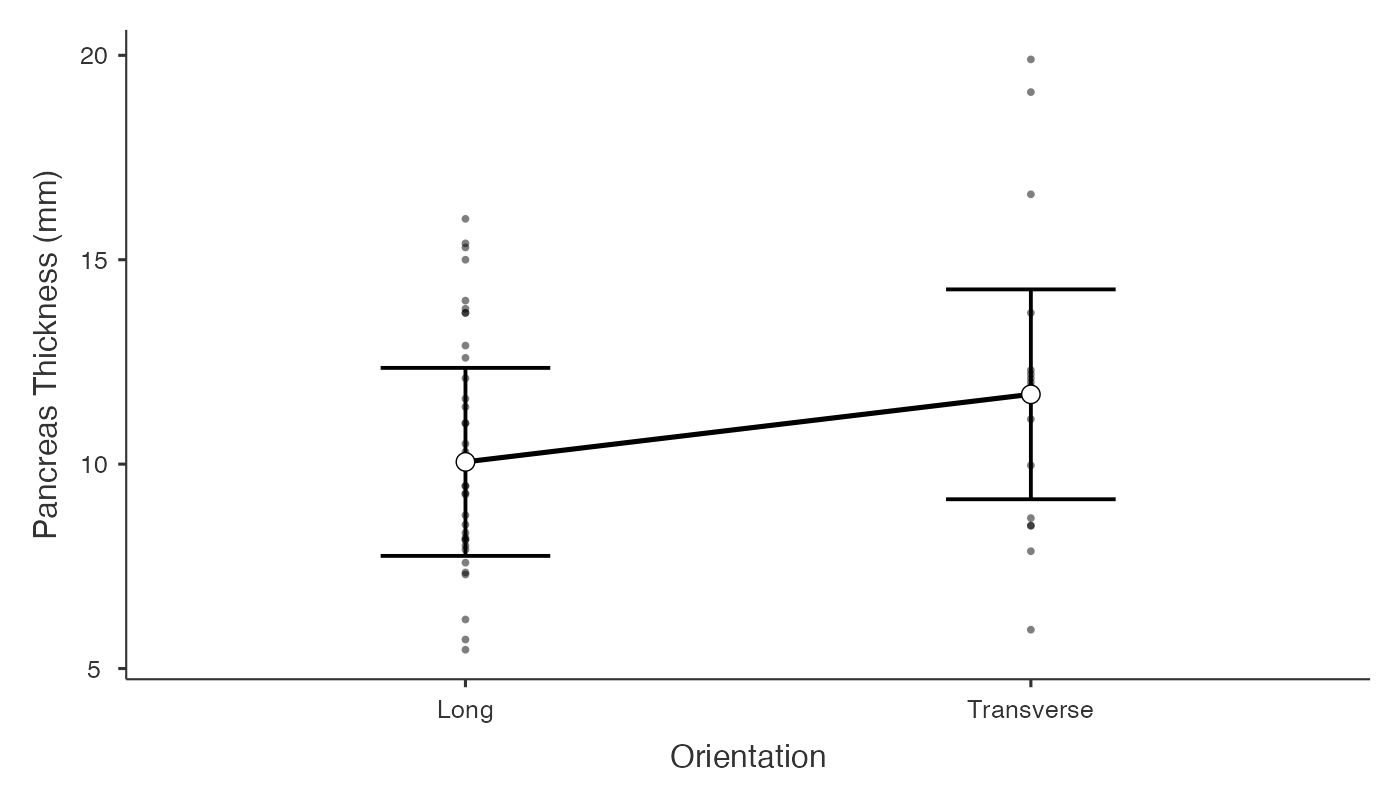


Points are observed values. Markers/line are model-estimated marginal means with 95% CI (adjusted for age, weight, BCS, VAT/SAT, sex and neuter status, diagnosis of HAC, and transducer frequency); Δ (transverse– longitudinal orientation) = *B* = 1.653mm (95% CI -0.081, 3.386), p = 0.062

Table 8: Sensitivity analysis for the transducer orientation–pancreatic thickness association in multivariable models.

| Model Substitutions | Unstandardised Coefficient (*B)* | Unstandardised Coefficient (*B)* 95% CI | p-value |
| --- | --- | --- | --- |
| BCS + complete VAT/SAT  (primary analysis) | 1.653 | -0.081, 3.386 | 0.062 |
| BCS + imputed VAT/SAT | 1.497 | 0.018, 2.976 | **0.047*** |
| BCS + complete VAT | 1.707 | 0.023, 3.391 | **0.047*** |
| BCS + imputed VAT | 1.518 | 0.061, 2.974 | **0.041*** |
| Complete TAT + complete VAT | 1.730 | 0.079, 3.381 | **0.040*** |
| Imputed TAT + imputed VAT | 1.500 | 0.025, 2.975 | **0.046*** |

*statistically significant with p < 0.05

Model: pancreas thickness = intercept + age + weight + substituted adiposity (BCS, complete-TAT, imputed-TAT) + substituted fat distribution (complete-VAT/SAT, imputed-VAT/SAT, complete-VAT, imputed-VAT) + sex + neuter status + HAC diagnosis + image orientation + transducer frequency.

1. Graph 9: Adjusted Predictions of Pancreatic Thickness by Transducers Frequency (95% CI)


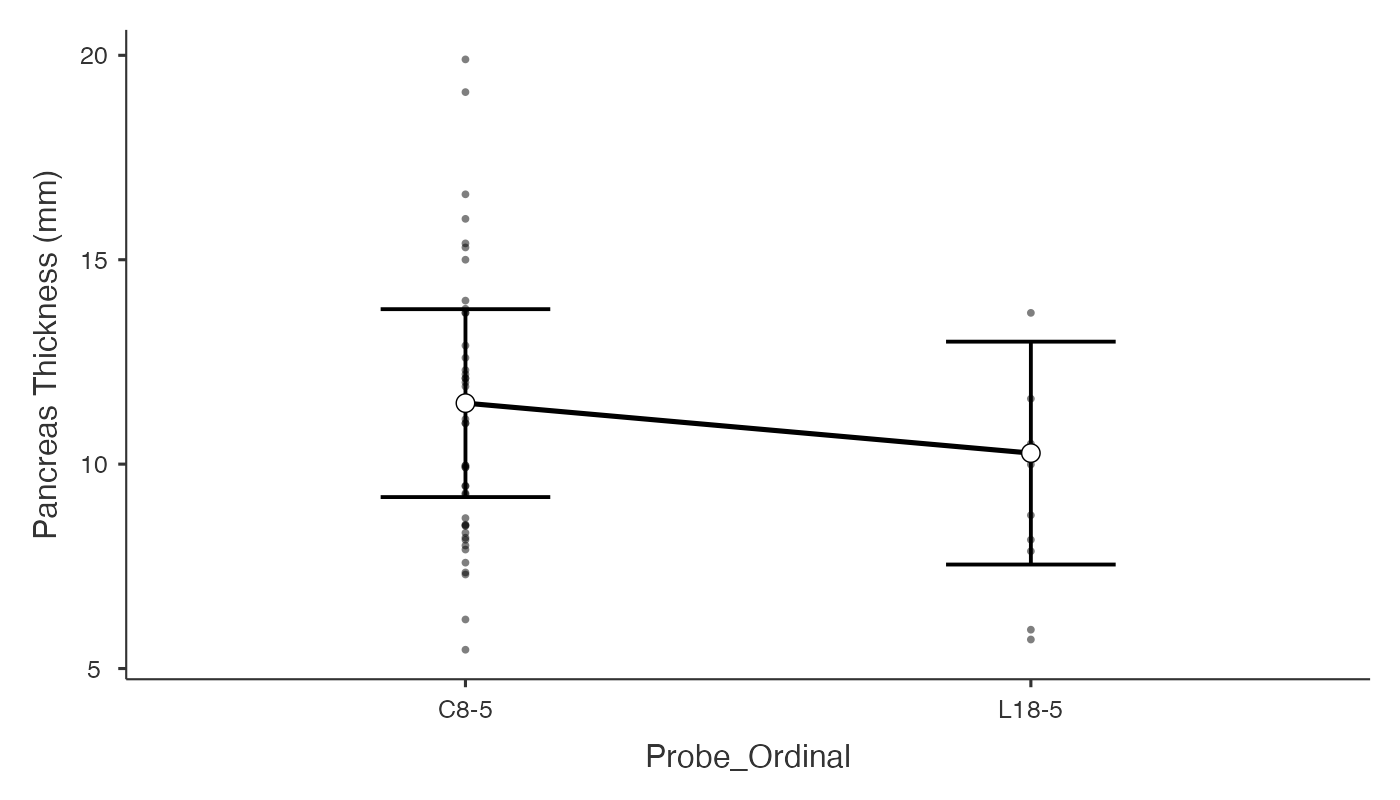


Points are observed values. Markers/line are model-estimated marginal means with 95% CI (adjusted for age, weight, BCS, imputed-VAT/SAT, sex and neuter status, diagnosis of HAC, and pancreas orientation); Δ(L18-5 – C8-5) = *B* = -1.222 mm (95% CI -3.366,0.921), p = 0.264

Table 9: Sensitivity analysis for the transducer frequency–pancreatic thickness association in multivariable models.

| Model Substitutions | Unstandardised Coefficient (*B)* | Unstandardised Coefficient (*B)* 95% CI | p-value |
| --- | --- | --- | --- |
| BCS + complete VAT/SAT  (primary analysis) | -1.222 | -3.366, 0.921 | 0.264 |
| BCS + imputed VAT/SAT | -1.427 | -3.431, 0.578 | 0.163 |
| BCS + complete VAT | -1.206 | -3.295, 0.882 | 0.258 |
| BCS + imputed VAT | -1.418 | -3.391, 0.556 | 0.159 |
| Complete TAT + complete VAT | -1.174 | -3.222, 0.875 | 0.261 |
| Imputed TAT + imputed VAT | -1.393 | -3.381, 0.595 | 0.170 |

Model: pancreas thickness = intercept + age + weight + substituted adiposity (BCS, complete-TAT, imputed-TAT) + substituted fat distribution (complete-VAT/SAT, imputed-VAT/SAT, complete-VAT, imputed-VAT) + sex + neuter status + HAC diagnosis + image orientation + transducer frequency.

**B. Quantitative Measures of Organ Echogenicity**

1. Table 10: Quantitative ultrasound pixel intensities of pancreas, liver, right renal cortex and spleen in 72 dogs.

|  | **N** | **Mean** | **Median** | **SD** | **IQR** | **Min.** | **Max.** | **Shapiro-Wilk** |
| --- | --- | --- | --- | --- | --- | --- | --- | --- |
| **Pancreas Absolute Pixel Intensity** | 72 | 65.6 | 60.1 | 23.1 | 22.7 | 32.6 | 137.0 | <0.001 |
| **Liver Absolute Pixel Intensity** | 67 | 55.6 | 49.1 | 23.2 | 26.4 | 15.1 | 117.2 | 0.001 |
| **Normal Liver Absolute Pixel Intensity** | 47 | 45.9 | 42.9 | 13.8 | 18.2 | 15.1 | 86.6 | 0.727 |
| **Right Kidney Absolute Pixel Intensity** | 69 | 51.1 | 49.5 | 18.8 | 21.5 | 14.4 | 110.8 | 0.251 |
| **Normal Right Kidney Absolute Pixel Intensity** | 60 | 50.3 | 48.2 | 17.7 | 20.9 | 14.4 | 110.8 | 0.084 |
| **Spleen Absolute Pixel Intensity** | 64 | 71.9 | 70.4 | 19.4 | 19.5 | 32.5 | 139.0 | 0.002 |
| **Normal Spleen Absolute Pixel Intensity** | 51 | 72.3 | 71.4 | 18.0 | 21.4 | 32.5 | 129.3 | 0.087 |
| **Pancreas/Liver Ratio** | 67 | 1.27 | 1.20 | 0.40 | 0.41 | 0.61 | 2.76 | <0.001 |
| **Pancreas/Normal Liver Ratio** | 47 | 1.37 | 1.26 | 0.40 | 0.35 | 0.86 | 2.76 | <0.001 |
| **Pancreas/Right Kidney Ratio** | 69 | 1.45 | 1.21 | 0.74 | 0.65 | 0.60 | 5.43 | <0.001 |
| **Pancreas/Normal Right Kidney Ratio** | 60 | 1.43 | 1.21 | 0.72 | 0.62 | 0.76 | 5.43 | <0.001 |
| **Pancreas/Spleen Ratio** | 64 | 0.93 | 0.90 | 0.26 | 0.29 | 0.49 | 1.92 | 0.005 |
| **Pancreas/ Normal Spleen Ratio** | 51 | 0.91 | 0.88 | 0.25 | 0.28 | 0.49 | 1.92 | 0.001 |

Freehand ROIs. Normal denotes organs judged subjectively normal on ultrasound and used for sensitivity analyses.

1. Table 11: Within-dog pairwise comparisons of pixel intensities between organs.

| **Organ 1** | **N** | **Organ 1 Median Pixel Intensity** | **Organ 2** | **Organ 2 Median Pixel Intensity** | **Effect Size (r_rb_)** | **Unadjusted p-value** |
| --- | --- | --- | --- | --- | --- | --- |
| **Pancreas** | 67 | 58.3 | Liver | 49.1 | 0.650 | **<0.001*** |
|  | 47 | 57.6 | Normal Liver | 42.9 | 0.922 | **<0.001*** |
| **Pancreas** | 69 | 60.0 | Right Kidney | 49.5 | 0.669 | **<0.001*** |
|  | 60 | 60.1 | Normal Right Kidney | 48.2 | 0.730 | **<0.001*** |
| **Pancreas** | 64 | 60.1 | Spleen | 70.5 | -0.446 | **0.002*** |
|  | 51 | 60.1 | Normal Spleen | 71.4 | -0.545 | **<0.001*** |
| **Liver** | 67 | 49.1 | Right Kidney | 48.2 | 0.245 | 0.082 |
| **Normal Liver** | 44 | 44.0 | Normal Right Kidney | 47.4 | -0.093 | 0.599 |
| **Liver** | 62 | 49.1 | Spleen | 71.0 | -0.809 | **<0.001*** |
| **Normal Liver** | 38 | 47.1 | Normal Spleen | 71.3 | -0.957 | **<0.001*** |
| **Right Kidney** | 63 | 48.2 | Spleen | 71.2 | -0.847 | **<0.001*** |
| **Normal Right Kidney** | 44 | 48.2 | Normal Spleen | 71.5 | -0.927 | **<0.001*** |

*statistically significant with p < 0.05

r_rb_ - rank-biserial

Wilcoxon signed-rank tests. Normal’ denotes organs judged subjectively normal on ultrasound and used for sensitivity analyses.

1. Table 12: Pancreatic absolute and relative intensity across subjective echogenicity groups.

| **Ultrasound Pixel Intensity Value** | **n** | **Hypoechoic**  **(A)** | **Isoechoic**  **(B)** | **Hyperechoic**  **(C)** | **Effect Size**  **(ε²)** | **DS–CF group*** | **Omnibus Unadjusted p-value** |
| --- | --- | --- | --- | --- | --- | --- | --- |
|  |  | Median (IQR)  [n] | Median (IQR)  [n] | Median (IQR)  [n] |  |  |  |
| **Pancreatic Absolute Pixel Intensity** | 72 | 54.34  (17.53)  [55] | 78.02  (33.60)  [11] | 106.20  (21.27)  [6] | 0.332 | A < B  A < C  B = C | <**0.001*** |
| **Pancreas/Liver Relative Pixel Intensity** | 67 | 1.194  (0.407)  [52] | 1.187  (0.266)  [9] | 1.385  (0.885)  [6] | 0.053 | A = B  A = C  B = C | 0.175 |
| **Pancreas/Normal Liver Relative Pixel Intensity** | 47 | 1.244  (0.323)  [40] | 1.401  (0.331) [4] | 2.269  (0.765)  [3] | 0.091 | A = B  A = C  B = C | 0.122 |
| **Pancreas/Right Kidney Relative Pixel Intensity** | 69 | 1.131  (0.509)  [52] | 1.324  (0.582)  [11] | 2.107  (0.166)  [6] | 0.147 | A = B  A < C  B < C | **0.007*** |
| **Pancreas/Normal Right Kidney Relative Pixel Intensity** | 60 | 1.137  (0.463)  [46] | 1.324  (0.582)  [9] | 2.159  (0.128)  [5] | 0.152 | A = B  A < C  B < C | **0.011*** |
| **Pancreas/Spleen Relative Pixel Intensity** | 64 | 0.870  (0.253)  [49] | 1.012  (0.273)  [10] | 1.413  (0.247)  [5] | 0.218 | A = B  A < C  B = C | **0.001*** |
| **Pancreas/Normal Spleen Relative Pixel Intensity** | 51 | 0.862  (0.238)  [39] | 1.062  (0.310)  [9] | 1.246  (0.509)  [3] | 0.190 | A < B  A = C  B = C | **0.009*** |

*statistically significant with p < 0.05

Kruskal–Wallis overall test with DSCF post-hoc pairwise comparisons. Normal’ denotes organs judged subjectively normal on ultrasound and used for sensitivity analyses

IQR - interquartile range DS–CF (Dwass–Steel–Critchlow–Fligner) pairwise comparisons.

- Within each row, symbols summarize adjusted pairwise results (α=0.05):
- “=” → no statistically significant difference between groups
- “<” → group on the left is lower than the group on the right (p<0.05)
- “>” → group on the left is higher than the group on the right (p<0.05)

1. Table 13. Sensitivity analysis of ordinal regression model of subjective pancreatic echogenicity (hypoechoic < isoechoic < hyperechoic) assessing patient characteristics and ultrasound acquisition factors in 72 dogs.

| **Predictor** |  | **OR** | **OR 95% CI** | **p-value** |
| --- | --- | --- | --- | --- |
| Age (years) | BCS + complete VAT/SAT  (primary analysis) | 1.146 | 0.927, 1.420 | 0.208 |
|  | BCS + imputed VAT/SAT | 1.134 | 0.929, 1.385 | 0.216 |
|  | BCS + complete VAT | 1.099 | 0.889, 1.360 | 0.383 |
|  | BCS + imputed VAT | 1.104 | 0.899, 1.355 | 0.344 |
|  | Complete TAT + complete VAT | 1.105 | 0.896, 1.360 | 0.352 |
|  | Imputed TAT + imputed VAT | 1.111 | 0.906, 1.364 | 0.311 |
| Weight (kg) | BCS + complete VAT/SAT  (primary analysis) | 0.995 | 0.901, 1.100 | 0.915 |
|  | BCS + imputed VAT/SAT | 1.053 | 0.980, 1.131 | 0.159 |
|  | BCS + complete VAT | 0.983 | 0.892, 1.080 | 0.730 |
|  | BCS + imputed VAT | 1.017 | 0.939, 1.101 | 0.680 |
|  | Complete TAT + complete VAT | 0.961 | 0.831, 1.110 | 0.593 |
|  | Imputed TAT + imputed VAT | 1.018 | 0.922, 1.124 | 0.720 |
| Total adiposity | BCS + complete VAT/SAT  (primary analysis) | 1.019 | 0.78, 1.800 | 0.949 |
|  | BCS + imputed VAT/SAT | 0.911 | 0.524, 1.582 | 0.739 |
|  | BCS + complete VAT | 0.822 | 0.432, 1.570 | 0.551 |
|  | BCS + imputed VAT | 0.752 | 0.411, 1.377 | 0.355 |
|  | Complete TAT + complete VAT | 1.125 | 0.579, 2.190 | 0.727 |
|  | Imputed TAT + imputed VAT | 0.967 | 0.570, 1.638 | 0.900 |
| Visceral adiposity | BCS + complete VAT/SAT  (primary analysis) | 1.011 | 0.788, 1.300 | 0.933 |
|  | BCS + imputed VAT/SAT | 1.106 | 0.889, 1.376 | 0.364 |
|  | BCS + complete VAT | 1.088 | 0.963, 1.230 | 0.177 |
|  | BCS + imputed VAT | 1.117 | 1.000, 1.248 | 0.050 |
|  | Complete TAT + complete VAT | 0955 | 0.505, 1.800 | 0.887 |
|  | Imputed TAT + imputed VAT | 1.125 | 0.684, 1.849 | 0.643 |
| Sex (Female vs Male) | BCS + complete VAT/SAT  (primary analysis) | 1.092 | 0.208, 5.720 | 0.917 |
|  | BCS + imputed VAT/SAT | 1.023 | 0.239, 4.383 | 0.976 |
|  | BCS + complete VAT | 1.087 | 0.198, 5.970 | 0.923 |
|  | BCS + imputed VAT | 1.230 | 0.275, 5.502 | 0.786 |
|  | Complete TAT + complete VAT | 1.444 | 0.217, 9.600 | 0.704 |
|  | Imputed TAT + imputed VAT | 1.408 | 0.283, 7.003 | 0.676 |
| Neuter status (Desexed vs entire) | BCS + complete VAT/SAT  (primary analysis) | 2.501 | 0.206, 30.34 | 0.472 |
|  | BCS + imputed VAT/SAT | 1.676 | 0.162, 17.340 | 0.665 |
|  | BCS + complete VAT | 3.386 | 0.243, 47.260 | 0.364 |
|  | BCS + imputed VAT | 2.441 | 0.214, 27.798 | 0.472 |
|  | Complete TAT + complete VAT | 4.307 | 0.343, 54.160 | 0.258 |
|  | Imputed TAT + imputed VAT | 3.189 | 0.300, 33.871 | 0.336 |
| HAC (Yes vs No) | BCS + complete VAT/SAT  (primary analysis) | 10.452 | 0.630, 173.490 | 0.102 |
|  | BCS + imputed VAT/SAT | 26.192 | 2.079, 329.885 | **0.012*** |
|  | BCS + complete VAT | 12.029 | 0.722, 200.460 | 0.083 |
|  | BCS + imputed VAT | 28.678 | 2.365, 347.819 | **0.008*** |
|  | Complete TAT + complete VAT | 9.251 | 0.675, 126.850 | 0.096 |
|  | Imputed TAT + imputed VAT | 20.239 | 1.849, 221.536 | **0.014*** |
| Pancreas Thickness (mm) | BCS + complete VAT/SAT  (primary analysis) | 0.742 | 0.541, 1.020 | 0.064 |
|  | BCS + imputed VAT/SAT | 0.630 | 0.456, 0.870 | **0.005*** |
|  | BCS + complete VAT | 0.762 | 0.554, 1.050 | 0.096 |
|  | BCS + imputed VAT | 0.657 | 0.476, 0.906 | **0.010*** |
|  | Complete TAT + complete VAT | 0.778 | 0.562, 1.080 | 0.131 |
|  | Imputed TAT + imputed VAT | 0.661 | 0.478, 0.912 | **0.012*** |
| Pancreas Orientation (Transverse - Longitudinal) | BCS + complete VAT/SAT  (primary analysis) | 1.454 | 0.310, 6.82 | 0.635 |
|  | BCS + imputed VAT/SAT | 2.586 | 0.660, 10.136 | 0.173 |
|  | BCS + complete VAT | 1.506 | 0.318, 7.120 | 0.606 |
|  | BCS + imputed VAT | 2.451 | 0.615, 9.763 | 0.204 |
|  | Complete TAT + complete VAT | 1.473 | 0.316, 6.870 | 0.622 |
|  | Imputed TAT + imputed VAT | 2.421 | 0.619, 9.463 | 0.204 |
| Transducer (C8-5 vs L18-5) | BCS + complete VAT/SAT  (primary analysis) | 1.673 | 0.293, 9.550 | 0.563 |
|  | BCS + imputed VAT/SAT | 1.323 | 0.227, 7.699 | 0.756 |
|  | BCS + complete VAT | 1.998 | 0.353, 11.320 | 0.434 |
|  | BCS + imputed VAT | 1.421 | 0.245, 8.232 | 0.695 |
|  | Complete TAT + complete VAT | 2.271 | 0.397, 13.000 | 0.357 |
|  | Imputed TAT + imputed VAT | 1.524 | 0.264, 8.802 | 0.638 |

*statistically significant with p < 0.05

Model: subjective echogenicity = intercept + age + weight + substituted adiposity (BCS, complete-TAT, imputed-TAT) + substituted fat distribution (complete-VAT/SAT, imputed-VAT/SAT, complete-VAT, imputed-VAT) + sex + neuter status + HAC diagnosis + pancreatic thickness + image orientation + transducer frequency.

1. Table 14. Sensitivity analysis of logistic regression model of subjective pancreatic echotexture (homogeneous vs heterogeneous) assessing patient characteristics and ultrasound acquisition factors in 72 dogs.

| **Predictor** |  | **OR** | **OR 95% CI** | **p-value** |
| --- | --- | --- | --- | --- |
| Age (years) | BCS + complete VAT/SAT  (primary analysis) | 1.251 | 1.030, 1.519 | **0.024*** |
|  | BCS + imputed VAT/SAT | 1.327 | 1.106, 1.591 | **0.002*** |
|  | BCS + complete VAT | 1.267 | 1.040, 1.542 | **0.019*** |
|  | BCS + imputed VAT | 1.327 | 1.104, 1.593 | **0.003*** |
|  | Complete TAT + complete VAT | 1.287 | 1.044, 1.587 | **0.018*** |
|  | Imputed TAT + imputed VAT | 1.361 | 1.119, 1.654 | **0.002*** |
| Weight (kg) | BCS + complete VAT/SAT  (primary analysis) | 0.980 | 0.896, 1.073 | 0.664 |
|  | BCS + imputed VAT/SAT | 1.015 | 0.945, 1.090 | 0.683 |
|  | BCS + complete VAT | 1.008 | 0.923, 1.100 | 0.864 |
|  | BCS + imputed VAT | 1.037 | 0.962, 1.118 | 0.346 |
|  | Complete TAT + complete VAT | 0.896 | 0.775, 1.035 | 0.135 |
|  | Imputed TAT + imputed VAT | 0.968 | 0.878, 1.067 | 0.510 |
| Total adiposity | BCS + complete VAT/SAT  (primary analysis) | 1.091 | 0.610, 1.951 | 0.769 |
|  | BCS + imputed VAT/SAT | 0.920 | 0.538, 1.573 | 0.761 |
|  | BCS + complete VAT | 1.106 | 0.597, 2.048 | 0.749 |
|  | BCS + imputed VAT | 0.880 | 0.513, 1.508 | 0.641 |
|  | Complete TAT + complete VAT | 1.906 | 1.066, 3.409 | **0.030*** |
|  | Imputed TAT + imputed VAT | 1.560 | 1.010, 2.411 | **0.045*** |
| Visceral adiposity | BCS + complete VAT/SAT  (primary analysis) | 0.836 | 0.627, 1.115 | 0.223 |
|  | BCS + imputed VAT/SAT | 0.823 | 0.646 1.049 | 0.116 |
|  | BCS + complete VAT | 0.927 | 0.808, 1.064 | 0.281 |
|  | BCS + imputed VAT | 0.938 | 0.842, 1.044 | 0.242 |
|  | Complete TAT + complete VAT | 0.514 | 0.292 0.903 | **0.021*** |
|  | Imputed TAT + imputed VAT | 0.619 | 0.409, 0.936 | **0.023*** |
| Sex (Female vs Male) | BCS + complete VAT/SAT  (primary analysis) | 2.542 | 0.572, 11.302 | 0.220 |
|  | BCS + imputed VAT/SAT | 2.190 | 0.596, 8.047 | 0.238 |
|  | BCS + complete VAT | 2.327 | 0.539, 10.042 | 0.258 |
|  | BCS + imputed VAT | 1.892 | 0.534, 6.702 | 0.323 |
|  | Complete TAT + complete VAT | 5.142 | 0.962, 27.495 | 0.056 |
|  | Imputed TAT + imputed VAT | 3.532 | 0.883, 14.134 | 0.074 |
| Neuter status (Desexed vs entire) | BCS + complete VAT/SAT  (primary analysis) | 0.604 | 0.038, 9.543 | 0.720 |
|  | BCS + imputed VAT/SAT | 0.818 | 0.095, 7.021 | 0.854 |
|  | BCS + complete VAT | 0.529 | 0.028, 9.870 | 0.670 |
|  | BCS + imputed VAT | 0.753 | 0.083, 6.822 | 0.800 |
|  | Complete TAT + complete VAT | 0.600 | 0.031, 11.440 | 0.734 |
|  | Imputed TAT + imputed VAT | 1.073 | 0.104 10.966 | 0.953 |
| HAC (Yes vs No) | BCS + complete VAT/SAT  (primary analysis) | 2.633 | 0.087, 79.690 | 0.578 |
|  | BCS + imputed VAT/SAT | 5.420 | 0.262, 112.196 | 0.274 |
|  | BCS + complete VAT | 2.183 | 0.090 53.178 | 0.632 |
|  | BCS + imputed VAT | 4.447 | 0.281, 70.222 | 0.289 |
|  | Complete TAT + complete VAT | 2.654 | 0.100, 70.730 | 0.560 |
|  | Imputed TAT + imputed VAT | 3.408 | 0.245, 47.474 | 0.362 |
| Pancreas Thickness (mm) | BCS + complete VAT/SAT  (primary analysis) | 1.109 | 0.869, 1.416 | 0.404 |
|  | BCS + imputed VAT/SAT | 1.025 | 0.825, 1.273 | 0.823 |
|  | BCS + complete VAT | 1.096 | 0.857, 1.402 | 0.463 |
|  | BCS + imputed VAT | 1.024 | 0.821, 1.276 | 0.836 |
|  | Complete TAT + complete VAT | 1.177 | 0.891, 1.556 | 0.251 |
|  | Imputed TAT + imputed VAT | 1.032 | 0.824, 1.293 | 0.784 |
| Pancreas Orientation (Transverse - Longitudinal) | BCS + complete VAT/SAT  (primary analysis) | 0.423 | 0.087, 2.048 | 0.285 |
|  | BCS + imputed VAT/SAT | 0.813 | 0.210, 3.149 | 0.765 |
|  | BCS + complete VAT | 0.364 | 0.075 1.756 | 0.208 |
|  | BCS + imputed VAT | 0.737 | 0.194, 2.804 | 0.654 |
|  | Complete TAT + complete VAT | 0.357 | 0.072, 1.763 | 0.206 |
|  | Imputed TAT + imputed VAT | 0.657 | 0.170, 2.542 | 0.543 |
| Transducer (C8-5 vs L18-5) | BCS + complete VAT/SAT  (primary analysis) | 0.080 | 0.007, 0.894 | **0.040*** |
|  | BCS + imputed VAT/SAT | 0.068 | 0.006, 0.791 | **0.032*** |
|  | BCS + complete VAT | 0.078 | 0.007, 0.880 | **0.039*** |
|  | BCS + imputed VAT | 0.071 | 0.006, 0.814 | **0.034*** |
|  | Complete TAT + complete VAT | 0.103 | 0.008, 1.283 | 0.077 |
|  | Imputed TAT + imputed VAT | 0.099 | 0.009, 1.107 | 0.060 |

*statistically significant with p < 0.05

Model: subjective echogenicity = intercept + age + weight + substituted adiposity (BCS, complete-TAT, imputed-TAT) + substituted fat distribution (complete-VAT/SAT, imputed-VAT/SAT, complete-VAT, imputed-VAT) + sex + neuter status + HAC diagnosis + pancreatic thickness + image orientation + transducer frequency.

Table 14. Sensitivity analysis of general linear model outcomes for pancreatic absolute pixel intensity and relative to splenic intensity

|  | |  |  | **Pancreas Absolute Pixel Intensity** | | | **Pancreas/Spleen Pixel Intensity Ratio** | | | **Pancreas/Liver Pixel Intensity Ratio** | | | **Pancreas/Right Kidney Pixel Intensity Ratio** | | |
| --- | --- | --- | --- | --- | --- | --- | --- | --- | --- | --- | --- | --- | --- | --- | --- |
|  | |  |  | **B** | **95% CI** | **p** | **B** | **95% CI** | **p** | **B** | **95% CI** | **p** | **B** | **95% CI** | **p** |
| Age (years) | | All Reference Organs | BCS + complete VAT/SAT  (primary analysis) | 1.695 | 0.216, 3.174 | **0.021*** | 0.017 | 0.000, 0.034 | **0.045*** | -0.020 | -0.045, 0.005 | 0.111 | 0.056 | -0.004, 0.117 | 0.067 |
|  | |  | BCS + imputed VAT/SAT | 1.166 | -0.241, 2.574 | 0.104 | 0.018 | 0.002, 0.034 | **0.027*** |  |  |  |  |  |  |
|  | |  | BCS + complete VAT | 1.386 | 0.028, 2.745 | **0.046*** | 0.014 | -0.002, 0.030 | 0.090 |  |  |  |  |  |  |
|  | |  | BCS + imputed VAT | 0.992 | -0.387, 2.372 | 0.159 | 0.014 | -0.002, 0.029 | 0.085 |  |  |  |  |  |  |
|  | |  | Complete TAT + complete VAT | 1.302 | -0.085, 2.689 | 0.066 | 0.016 | 0.000, 0.032 | 0.059 |  |  |  |  |  |  |
|  | |  | Imputed TAT + imputed VAT | 1.068 | -0.330, 2.467 | 0.134 | 0.015 | 0.000, 0.031 | 0.062 |  |  |  |  |  |  |
|  | |  |  |  |  |  |  |  |  |  |  |  |  |  |  |
| Weight (kg) | | All Reference Organs | BCS + complete VAT/SAT  (primary analysis) | -0.256 | -1.009, 0.497 | 0.493 | 0.004 | -0.006, 0.013 | 0.443 | -0.004 | -0.017, 0.009 | 0.561 | -0.009 | -0.040, 0.022 | 0.555 |
|  | |  | BCS + imputed VAT/SAT | -0.084 | -0.733, 0.565 | 0.799 | 0.010 | 0.003, 0.017 | **0.007*** |  |  |  |  |  |  |
|  | |  | BCS + complete VAT | -0.634 | -1.3447, 0.0758 | 0.080 | 0.000 | -0.008, 0.009 | 0.935 |  |  |  |  |  |  |
|  | |  | BCS + imputed VAT | -0.544 | -1.212, 0.124 | 0.110 | 0.006 | -0.002, 0.013 | 0.132 |  |  |  |  |  |  |
|  | |  | Complete TAT + complete VAT | -0.411 | -1.2886", 0.468 | 0.359 | -0.005 | -0.015, 0.005 | 0.324 |  |  |  |  |  |  |
|  | |  | Imputed TAT + imputed VAT | -0.588 | -1.411, 0.235 | 0.161 | 0.001 | -0.009, 0.010 | 0.890 |  |  |  |  |  |  |
|  | |  |  |  |  |  |  |  |  |  |  |  |  |  |  |
| Total adiposity | |  | BCS + complete VAT/SAT  (primary analysis) | -2.125 | -6.738, 2.487 | 0.353 | -0.011 | -0.065, 0.044 | 0.700 | 0.052 | -0.028, 0.132 | 0.205 | 0.025 | -0.168, 0.219 | 0.797 |
|  | |  | BCS + imputed VAT/SAT | -2.555 | -7.113, 2.004 | 0.272 | -0.039 | -0.090, 0.012 | 0.132 |  |  |  |  |  |  |
|  | |  | BCS + complete VAT | -3.543 | -8.0729, 0.9867 | 0.125 | -0.033 | -0.087, 0.021 | 0.225 |  |  |  |  |  |  |
|  | |  | BCS + imputed VAT | -3.330 | -7.815, 1.154 | 0.145 | -0.057 | -0.105, -0.008 | **0.023*** |  |  |  |  |  |  |
|  | |  | Complete TAT + complete VAT | -1.962 | -5.0643, 1.141 | 0.215 | 0.024 | -0.011, 0.059 | 0.173 |  |  |  |  |  |  |
|  | |  | Imputed TAT + imputed VAT | -0.446 | -3.959, 3.068 | 0.804 | 0.022 | -0.017, 0.060 | 0.271 |  |  |  |  |  |  |
|  | |  |  |  |  |  |  |  |  |  |  |  |  |  |  |
| Visceral adiposity | | All Reference Organs | BCS + complete VAT/SAT  (primary analysis) | 1.818 | -0.430, 4.066 | 0.103 | 0.011 | -0.015, 0.036 | 0.408 | -0.034 | -0.072, 0.003 | 0.070 | -0.046 | -0.136, 0.044 | 0.313 |
|  | |  | BCS + imputed VAT/SAT | 2.631 | 0.613, 4.650 | **0.011*** | 0.016 | -0.007, 0.038 | 0.170 |  |  |  |  |  |  |
|  | |  | BCS + complete VAT | 1.332 | 0.3082, 2.3554 | **0.011*** | 0.015 | 0.003, 0.027 | **0.017*** |  |  |  |  |  |  |
|  | |  | BCS + imputed VAT | 1.502 | 0.594, 2.411 | **0.001*** | 0.015 | 0.005, 0.025 | **0.003*** |  |  |  |  |  |  |
|  | |  | Complete TAT + complete VAT | 2.835 | -0.2828, 5.952 | 0.075 | -0.012 | -0.046, 0.023 | 0.502 |  |  |  |  |  |  |
|  | |  | Imputed TAT + imputed VAT | 1.622 | -1.703, 4.948 | 0.339 | -0.009 | -0.045, 0.027 | 0.620 |  |  |  |  |  |  |
|  | |  |  |  |  |  |  |  |  |  |  |  |  |  |  |
| Sex (Female vs Male) | | All Reference Organs | BCS + complete VAT/SAT  (primary analysis) | 4.115 | -8.504, 16.733 | 0.511 | -0.084 | -0.232, 0.064 | 0.264 | -0.032 | -0.242, 0.178 | 0.765 | -0.069 | -0.576, 0.438 | 0.789 |
|  | |  | BCS + imputed VAT/SAT | -0.201 | -11.664, 11.261 | 0.973 | -0.094 | -0.226, 0.038 | 0.164 |  |  |  |  |  |  |
|  | |  | BCS + complete VAT | 5.675 | -5.9668, 17.316 | 0.339 | -0.067 | -0.206, 0.072 | 0.345 |  |  |  |  |  |  |
|  | |  | BCS + imputed VAT | 1.978 | -9.120, 13.077 | 0.727 | -0.079 | -0.203, 0.044 | 0.206 |  |  |  |  |  |  |
|  | |  | Complete TAT + complete VAT | 3.257 | -9.654, 16.169 | 0.621 | -0.017 | -0.166, 0.132 | 0.821 |  |  |  |  |  |  |
|  | |  | Imputed TAT + imputed VAT | 3.312 | -8.546, 15.17 | 0.584 | -0.020 | -0.155, 0.116 | 0.777 |  |  |  |  |  |  |
|  | |  |  |  |  |  |  |  |  |  |  |  |  |  |  |
| Neuter status (Desexed vs entire) | | All Reference Organs | BCS + complete VAT/SAT  (primary analysis) | 3.076 | -18.130, 24.281 | 0.770 | -0.162 | -0.399, 0.075 | 0.181 | -0.336 | -0.685, 0.013 | 0.059 | -0.343 | -1.183, 0.496 | 0.423 |
|  | |  | BCS + imputed VAT/SAT | 0.038 | -18.569, 18.646 | 0.997 | -0.151 | -0.350, 0.048 | 0.138 |  |  |  |  |  |  |
|  | |  | BCS + complete VAT | 6.889 | -13.494, 27.2721 | 0.508 | -0.101 | -0.330, 0.128 | 0.388 |  |  |  |  |  |  |
|  | |  | BCS + imputed VAT | 4.047 | -14.393, 22.488 | 0.667 | -0.099 | -0.290, 0.092 | 0.310 |  |  |  |  |  |  |
|  | |  | Complete TAT + complete VAT | 6.040 | -14.617, 26.697 | 0.567 | -0.076 | -0.305, 0.154 | 0.518 |  |  |  |  |  |  |
|  | |  | Imputed TAT + imputed VAT | 5.405 | -13.436, 24.245 | 0.574 | -0.047 | -0.247, 0.153 | 0.645 |  |  |  |  |  |  |
|  | |  |  |  |  |  |  |  |  |  |  |  |  |  |  |
| HAC (Yes vs No) | | All Reference Organs | BCS + complete VAT/SAT  (primary analysis) | 6.572 | -18.472, 31.615 | 0.597 | 0.017 | -0.264, 0.298 | 0.906 | -0.108 | -0.521, 0.305 | 0.608 | 0.078 | -0.920, 1.076 | 0.878 |
|  | |  | BCS + imputed VAT/SAT | 8.765 | -13.384, 30.914 | 0.438 | 0.153 | -0.084, 0.391 | 0.206 |  |  |  |  |  |  |
|  | |  | BCS + complete VAT | 8.588 | -14.512, 31.6892 | 0.466 | 0.017 | -0.245, 0.278 | 0.901 |  |  |  |  |  |  |
|  | |  | BCS + imputed VAT | 10.029 | -11.388, 31.446 | 0.359 | 0.156 | -0.067, 0.379 | 0.169 |  |  |  |  |  |  |
|  | |  | Complete TAT + complete VAT | 1.668 | -20.271, 23.607 | 0.882 | -0.035 | -0.277, 0.207 | 0.778 |  |  |  |  |  |  |
|  | |  | Imputed TAT + imputed VAT | 4.913 | -15.725, 25.551 | 0.641 | 0.070 | -0.147, 0.287 | 0.526 |  |  |  |  |  |  |
|  | |  |  |  |  |  |  |  |  |  |  |  |  |  |  |
| Pancreas Thickness (mm) | | All Reference Organs | BCS + complete VAT/SAT  (primary analysis) | -0.101 | -2.134, 1.932 | 0.920 | -0.005 | -0.029, 0.019 | 0.681 | 0.009 | -0.026, 0.044 | 0.622 | -0.018 | -0.103, 0.067 | 0.677 |
|  | |  | BCS + imputed VAT/SAT | -0.864 | -2.755, 1.029 | 0.371 | -0.017 | -0.038, 0.004 | 0.111 |  |  |  |  |  |  |
|  | |  | BCS + complete VAT | 0.110 | -1.801, 2.020 | 0.910 | -0.003 | -0.026, 0.020 | 0.794 |  |  |  |  |  |  |
|  | |  | BCS + imputed VAT | -0.557 | -2.420, 1.305 | 0.558 | -0.012 | -0.033, 0.008 | 0.227 |  |  |  |  |  |  |
|  | |  | Complete TAT + complete VAT | 0.004 | -1.957, 1.964 | 0.997 | 0.003 | -0.020, 0.026 | 0.783 |  |  |  |  |  |  |
|  | |  | Imputed TAT + imputed VAT | -0.452 | -2.340, 1.436 | 0.639 | -0.010 | -0.031, 0.011 | 0.353 |  |  |  |  |  |  |
|  | |  |  |  |  |  |  |  |  |  |  |  |  |  |  |
| Pancreas Orientation (Transverse - Longitudinal) | | All Reference Organs | BCS + complete VAT/SAT  (primary analysis) | 0.974 | -11.677, 13.624 | 0.877 | 0.104 | -0.047, 0.256 | 0.178 | 0.076 | -0.153, 0.305 | 0.516 | 0.338 | -0.204, 0.880 | 0.221 |
|  | |  | BCS + imputed VAT/SAT | 1.936 | -9.658, 13.529 | 0.744 | 0.184 | 0.053, 0.315 | **0.006*** |  |  |  |  |  |  |
|  | |  | BCS + complete VAT | 1.628 | -10.0925, 13.3482 | 0.785 | 0.100 | -0.042, 0.242 | 0.168 |  |  |  |  |  |  |
|  | |  | BCS + imputed VAT | 1.510 | -9.747, 12.768 | 0.793 | 0.171 | 0.048, 0.295 | **0.006*** |  |  |  |  |  |  |
|  | |  | Complete TAT + complete VAT | 1.408 | -10.411, 13.227 | 0.815 | 0.066 | -0.073, 0.206 | 0.351 |  |  |  |  |  |  |
|  | |  | Imputed TAT + imputed VAT | 1.306 | -10.237, 12.848 | 0.825 | 0.143 | 0.014, 0.272 | **0.030*** |  |  |  |  |  |  |
|  | |  |  |  |  |  |  |  |  |  |  |  |  |  |  |
| Transducer  (C9-2, C8-5 vs L18-5) | All Reference Organs | BCS + complete VAT/SAT  (primary analysis) | 9.167 | -6.115, 24.449 | 0.227 | 0.125 | -0.047, 0.297 | 0.155 | 0.052 | -0.199, 0.302 | 0.686 | -0.316 | -0.923, 0.290 | 0.306 |  |
|  |  | BCS + imputed VAT/SAT | 7.072 | -8.404, 22.549 | 0.370 | 0.096 | -0.073, 0.264 | 0.266 |  |  |  |  |  |  |  |
|  |  | BCS + complete VAT | 9.605 | -4.541, 23.751 | 0.183 | 0.137 | -0.025, 0.298 | 0.097 |  |  |  |  |  |  |  |
|  |  | BCS + imputed VAT | 7.013 | -7.988, 22.014 | 0.360 | 0.107 | -0.051, 0.265 | 0.185 |  |  |  |  |  |  |  |
|  |  | Complete TAT + complete VAT | 10.067 | -4.171, 24.305 | 0.166 | 0.153 | -0.008, 0.313 | 0.062 |  |  |  |  |  |  |  |
|  |  | Imputed TAT + imputed VAT | 7.348 | -7.963, 22.66 | 0.347 | 0.126 | -0.038, 0.291 | 0.133 |  |  |  |  |  |  |  |
