# Supplementary material for: Effect of Body Composition and Age on the Subjective and Quantitative Ultrasound Appearance of the Dogs’ Pancreas
Source: Vet Radiol Ultrasound. 2026 Jul 15;67(4):e70208. doi: 10.1111/vru.70208 (PMC13371151; doi:10.1111/vru.70208)
Supplement: Supplementary file 3 — vru70208‐Supp‐0003‐SuppMat3.docx [file VRU-67-0-s004.docx]

S3. Descriptive Statistics of Subjective Ultrasound Measures of the Canine Pancreas (Echogenicity, Echotexture)

1. Contingency Table: Subjective Classification of Echogenicity and Echotexture of Dogs Pancreases

|  | **Echotexture** | |  |  |
| --- | --- | --- | --- | --- |
| **Echogenicity** | **Homogeneous**  **N (proportion)** | **Heterogeneous**  **N (proportion)** | **Total**  **N (proportion)** | Fisher–Freeman–Halton exact test, p = 0.293; Cramér’s V = 0.186 |
| Hypoechoic | 31 (0.76) | 24 (0.77) | 55 (0.76) | Logistic Trend OR = 1.174, [95% CI: 0.556, 2.480], p = 0.674 |
| Isoechoic | 8 (0.20) | 3 (0.10) | 11 (0.15) |  |
| Hyperechoic | 2 (0.05) | 4 (0.13) | 6 (0.08) |  |
| Total | 41 (0.57) | 31 (0.43) | 72 |  |

1. Bar Graph: Frequencies of Echogenicity Within Echotexture Groups.
