# Supplementary material for: Effect of Body Composition and Age on the Subjective and Quantitative Ultrasound Appearance of the Dogs’ Pancreas
Source: Vet Radiol Ultrasound. 2026 Jul 15;67(4):e70208. doi: 10.1111/vru.70208 (PMC13371151; doi:10.1111/vru.70208)
Supplement: Supplementary file 4 — vru70208‐Supp‐0004‐SuppMat4.docx [file VRU-67-0-s003.docx]

S4. Validation of Quantitative Measures of Echogenicity and Echotexture

Correlation of variables are summarised in table xxx. Dependent variables with correlations above |0.80| included between first order and secondary order GLCM measures (conventional standard deviation, discretised standard deviation, discretised entropy, and discretised energy), and bet

Texture variables that distinguished subjective echotexture (homogeneous vs heterogeneous) underwent discrimination, redundancy, and stability analyses to reduce multicollinearity and preserve interpretability of the principal component analysis (PCA). Discrimination of heterogeneous vs homogeneous echotexture was assessed using the Mann–Whitney U test with rank-biserial effect size, and receiver operator curve (ROC) area under the curve (AUC) (95% CI). False discovery rate was controlled at 5% using the Benjamini–Hochberg (BH) procedure (FDR-adjusted p-values or q-values). Texture variables with Pearson’s correlations exceeding |0.80| were deemed redundant and a single representative variable used for analysis. Sensitivity analysis selected final variables if comparable discretisation (bins 10/d1, bins 10/d10, and bins 5/d5) had differences AUC (|ΔAUC|) less than 0.05 and a non-significant paired DeLong test (20).

Table 1: Group discrimination of homogeneous and heterogeneous pancreatic echotexture using quantitative texture features (Mann-Whitney U Test)

|  | **Statistic** | **p** |  | **Effect Size** |
| --- | --- | --- | --- | --- |
| **CONVENTIONAL_std (3)** | 224 | **<.001** | Rank biserial correlation | 0.6475 |
| **DISCRETIZED_std (3)** | 262 | **<.001** | Rank biserial correlation | 0.5877 |
| **DISCRETIZED_HISTO_Entropy_log10 (3)** | 292 | **<.001** | Rank biserial correlation | 0.5405 |
| **DISCRETIZED_HISTO_Energy[=Uniformity] (3)** | 293 | **<.001** | Rank biserial correlation | -0.5389 |
| **GLCM_Homogeneity[=InverseDifference] (3)** | 498 | 0.120 | Rank biserial correlation | -0.2164 |
| **GLCM_Energy[=AngularSecondMoment] (3)** | 364 | **0.002** | Rank biserial correlation | -0.4272 |
| **GLCM_Contrast[=Variance] (3)** | 458 | **0.044** | Rank biserial correlation | 0.2793 |
| **GLCM_Correlation (3)** | 402 | **0.007** | Rank biserial correlation | 0.3674 |
| **GLCM_Entropy_log10 (3)** | 341 | **<.001** | Rank biserial correlation | 0.4634 |
| **GLCM_Dissimilarity (3)** | 487 | 0.092 | Rank biserial correlation | 0.2337 |
| **GLRLM_SRE (3)** | 459 | **0.045** | Rank biserial correlation | 0.2777 |
| **GLRLM_LRE (3)** | 473 | 0.065 | Rank biserial correlation | -0.2557 |
| **GLRLM_LGRE (3)** | 627 | 0.928 | Rank biserial correlation | 0.0134 |
| **GLRLM_HGRE (3)** | 570 | 0.462 | Rank biserial correlation | 0.1031 |
| **GLRLM_SRLGE (3)** | 599 | 0.684 | Rank biserial correlation | 0.0574 |
| **GLRLM_SRHGE (3)** | 507 | 0.146 | Rank biserial correlation | 0.2022 |
| **GLRLM_LRLGE (3)** | 578 | 0.519 | Rank biserial correlation | -0.0905 |
| **GLRLM_LRHGE (3)** | 592 | 0.627 | Rank biserial correlation | -0.0685 |
| **GLRLM_GLNU (3)** | 515 | 0.173 | Rank biserial correlation | 0.1896 |
| **GLRLM_RLNU (3)** | 355 | **0.001** | Rank biserial correlation | 0.4414 |
| **GLRLM_RP (3)** | 491 | 0.102 | Rank biserial correlation | 0.2274 |
| **NGLDM_Coarseness (3)** | 544 | 0.303 | Rank biserial correlation | -0.1440 |
| **NGLDM_Contrast (3)** | 344 | **<.001** | Rank biserial correlation | 0.4587 |
| **NGLDM_Busyness (3)** | 575 | 0.497 | Rank biserial correlation | 0.0952 |
| **GLZLM_SZE (3)** | 430 | **0.019** | Rank biserial correlation | 0.3234 |
| **GLZLM_LZE (3)** | 505 | 0.140 | Rank biserial correlation | -0.2054 |
| **GLZLM_LGZE (3)** | 596 | 0.659 | Rank biserial correlation | -0.0622 |
| **GLZLM_HGZE (3)** | 538 | 0.272 | Rank biserial correlation | 0.1534 |
| **GLZLM_SZLGE (3)** | 606 | 0.743 | Rank biserial correlation | 0.0464 |
| **GLZLM_SZHGE (3)** | 443 | **0.028** | Rank biserial correlation | 0.3029 |
| **GLZLM_LZLGE (3)** | 549 | 0.330 | Rank biserial correlation | -0.1361 |
| **GLZLM_LZHGE (3)** | 513 | 0.166 | Rank biserial correlation | -0.1928 |
| **GLZLM_GLNU (3)** | 434 | **0.022** | Rank biserial correlation | 0.3171 |
| **GLZLM_ZLNU (3)** | 379 | **0.003** | Rank biserial correlation | 0.4036 |
| **GLZLM_ZP (3)** | 467 | 0.056 | Rank biserial correlation | 0.2651 |

 * statistically significant with p < 0.05 were included in redundancy analysis

Table 2. Pearsons (*r)* correlation between significant textural features discriminating pancreas heterogeneity to assess for redundancy of variable

|  | **CONVENTIONAL_std (3)** | **DISCRETIZED_std (3)** | **DISCRETIZED_HISTO_Entropy_log10 (3)** | **DISCRETIZED_HISTO_Energy[=Uniformity] (3)** | **GLCM_Energy[=AngularSecondMoment] (3)** | **GLCM_Contrast[=Variance] (3)** | **GLCM_Correlation (3)** | **GLCM_Entropy_log10 (3)** | **GLRLM_SRE (3)** | **GLRLM_RLNU (3)** | **NGLDM_Contrast (3)** | **GLZLM_SZE (3)** | **GLZLM_SZHGE (3)** | **GLZLM_GLNU (3)** | **GLZLM_ZLNU (3)** |
| --- | --- | --- | --- | --- | --- | --- | --- | --- | --- | --- | --- | --- | --- | --- | --- |
| **CONVENTIONAL_std (3)** | — |  |  |  |  |  |  |  |  |  |  |  |  |  |  |
| **DISCRETIZED_std (3)** | 0.870 | — |  |  |  |  |  |  |  |  |  |  |  |  |  |
| **DISCRETIZED_HISTO_Entropy_log10 (3)** | 0.897 | 0.959 | — |  |  |  |  |  |  |  |  |  |  |  |  |
| **DISCRETIZED_HISTO_Energy[=Uniformity] (3)** | -0.870 | -0.897 | -0.978 | — |  |  |  |  |  |  |  |  |  |  |  |
| **GLCM_Energy[=AngularSecondMoment] (3)** | -0.705 | -0.783 | -0.841 | 0.829 | — |  |  |  |  |  |  |  |  |  |  |
| **GLCM_Contrast[=Variance] (3)** | -0.026 | 0.442 | 0.324 | -0.242 | -0.418 | — |  |  |  |  |  |  |  |  |  |
| **GLCM_Correlation (3)** | 0.325 | 0.060 | 0.144 | -0.232 | 0.203 | -0.677 | — |  |  |  |  |  |  |  |  |
| **GLCM_Entropy_log10 (3)** | 0.659 | 0.857 | 0.853 | -0.797 | -0.944 | 0.643 | -0.321 | — |  |  |  |  |  |  |  |
| **GLRLM_SRE (3)** | 0.432 | 0.398 | 0.427 | -0.382 | -0.799 | 0.239 | -0.442 | 0.713 | — |  |  |  |  |  |  |
| **GLRLM_RLNU (3)** | 0.486 | 0.607 | 0.582 | -0.549 | -0.568 | 0.316 | 0.053 | 0.599 | 0.348 | — |  |  |  |  |  |
| **NGLDM_Contrast (3)** | 0.080 | 0.040 | 0.067 | -0.046 | -0.344 | 0.237 | -0.643 | 0.333 | 0.595 | -0.146 | — |  |  |  |  |
| **GLZLM_SZE (3)** | 0.245 | 0.254 | 0.219 | -0.146 | -0.577 | 0.326 | -0.671 | 0.556 | 0.858 | 0.177 | 0.713 | — |  |  |  |
| **GLZLM_SZHGE (3)** | 0.428 | 0.527 | 0.506 | -0.392 | -0.626 | 0.431 | -0.495 | 0.693 | 0.655 | 0.354 | 0.467 | 0.694 | — |  |  |
| **GLZLM_GLNU (3)** | 0.449 | 0.438 | 0.450 | -0.444 | -0.502 | 0.073 | 0.139 | 0.465 | 0.416 | 0.937 | -0.104 | 0.233 | 0.289 | — |  |
| **GLZLM_ZLNU (3)** | 0.597 | 0.635 | 0.614 | -0.561 | -0.621 | 0.238 | 0.006 | 0.646 | 0.508 | 0.900 | 0.046 | 0.391 | 0.536 | 0.916 | — |

Correlations exceeding |0.80| were deemed redundant and a single representative variable used for sensitivity analysis

Table 3: Significant Echotexture Features Discriminating Pancreas Heterogeneity Identified by Mann-Whitney U Test after False Discovery Rate Correction (Benjamini-Hochberg, 5% Significance; n=72; Bin10/d1) with Sensitivity Across Settings (AUC) for Paired Analysis (n=48)

|  | **Discrimination** | | | | **Sensitivity Analysis** | | | | |
| --- | --- | --- | --- | --- | --- | --- | --- | --- | --- |
| **Feature** | **Non-adjusted p-value** | **FDR**  **q-value** | **r_rb_** | **bin10/d1 (3)**  **AUC (s.e.)** | **bin10/d1 (3)**  **AUC (s.e.)** | **bin10/d10 (2)**  **AUC (s.e.)** | **bin5/d5 (1)**  **AUC (s.e.)** | **Max**  **\|Δ AUC\|** | **DeLong Test**  **Min p value** |
| Number of cases | 74 | | | | 48 | 48 | 48 |  |  |
| CONVENTIONAL_std (3) | <0.001 | **0.002*** | 0.648 | 0.824 (0.047) | 0.880 (0.048) | 0.880 (0.048) | 0.880 (0.048) | **0.000^#^** | **1.000^#^** |
| GLCM_Contrast (3) | 0.044 | **0.045*** | 0.279 | 0.640 (0.066) | 0.713 (0.075) | 0.839 (0.059) | 0.774 (0.067) | 0.126 | 0.011 |
| GLCM_Correlation (3) | 0.007 | **0.011*** | 0.367 | 0.684 (0.066) | 0.676 (0.080) | 0.515 (0.084) | 0.596 (0.083) | 0.161 | 0.011 |
| GLCM_Entropy_log10 (3) | <0.001 | **0.002*** | 0.463 | 0.732 (0.059) | 0.776 (0.068) | 0.870 (0.052) | 0.841 (0.057) | 0.094 | 0.024 |
| GLRLM_SRE (3) | 0.045 | **0.045*** | 0.278 | 0.639 (0.067) | 0.700 (0.079) | 0.693 (0.078) | 0.689 (0.078) | **0.011^#^** | **0.598^#^** |
| GLRLM_RLNU (3) | 0.001 | **0.002*** | 0.441 | 0.721 (0.061) | 0.776 (0.067) | 0.807 (0.062) | 0.785 (0.068) | **0.031^#^** | **0.289^#^** |
| NGLDM_Contrast (3) | <0.001 | **0.002*** | 0.459 | 0.729 (0.060) | 0.717 (0.078) | 0.691 (0.079) | 0.722 (0.075) | **0.031^#^** | **0.212^#^** |
| GLZLM_SZHGE (3) | 0.028 | **0.037*** | 0.303 | 0.651 (0.066) | 0.735 (0.075) | 0.761 (0.072) | 0.709 (0.079) | 0.052 | 0.188 |

AUC – Area under the curve; FDR - False Discovery Rate; r_rb_ - rank-biserial.

*Statistical difference for

DeLong’s test for pairwise AUC comparison and indicates insensitivity of test

Table 6. General linear model for univariate quantitative features of echotexture

|  |  | **Age (yr)** | **Weight (kg)** | **BCS** | **VAT/SAT** | **SEX** | **Neuter Status** | **HAC** | **Pancreas Thickness (mm)** | **Orientation** | **Probe** |
| --- | --- | --- | --- | --- | --- | --- | --- | --- | --- | --- | --- |
| **Conventional_std** | B | 0.174 | -0.031 | -0.299 | 0.058 | 0.126 | 0.141 | 1.709 | 0.007 | -0.209 | -4.040 |
|  | 95%CI | -0.048 | -0.137 | -1.033 | -0.297 | -1.690 | -2.743 | -1.839 | -0.288 | -2.038 | -6.424 |
|  |  | 0.396 | 0.076 | 0.436 | 0.413 | 1.942 | 3.025 | 5.256 | 0.302 | 1.619 | -1.656 |
|  | p | 0.125 | 0.572 | 0.426 | 0.749 | 0.892 | 0.924 | 0.345 | 0.962 | 0.822 | **<0.001** |
| **Discretised_std** | B | 0.027 | 0.007 | -0.056 | 0.018 | 0.019 | 0.020 | 0.194 | -0.006 | -0.034 | -0.212 |
|  | 95%CI | 0.001 | -0.005 | -0.141 | -0.023 | -0.190 | -0.313 | -0.215 | -0.040 | -0.244 | -0.486 |
|  |  | 0.053 | 0.020 | 0.029 | 0.059 | 0.229 | 0.352 | 0.603 | 0.028 | 0.177 | 0.063 |
|  | p | **0.039** | 0.243 | 0.194 | 0.397 | 0.856 | 0.908 | 0.352 | 0.713 | 0.754 | 0.131 |
| **Discretised_Entropy_Log10** | B | 0.005 | 0.001 | -0.005 | 0.002 | 0.010 | 0.002 | 0.039 | 0.000 | -0.004 | -0.056 |
|  | 95%CI | < - 0.001 | -0.002 | -0.023 | -0.006 | -0.034 | -0.068 | -0.047 | -0.007 | -0.049 | -0.113 |
|  |  | 0.010 | 0.003 | 0.012 | 0.011 | 0.053 | 0.071 | 0.124 | 0.007 | 0.040 | 0.002 |
|  | p | 0.067 | 0.640 | 0.545 | 0.595 | 0.667 | 0.966 | 0.375 | 0.930 | 0.842 | 0.056 |
| **Discretised_Energy** | B | -0.002 | < - 0.001 | < - 0.001 | < - 0.001 | -0.006 | -0.002 | -0.009 | < - 0.001 | 0.003 | 0.026 |
|  | 95%CI | -0.004 | -0.001 | -0.007 | -0.004 | -0.023 | -0.029 | -0.043 | -0.003 | -0.014 | 0.003 |
|  |  | 0.000 | 0.001 | 0.007 | 0.003 | 0.011 | 0.026 | 0.024 | 0.002 | 0.021 | 0.049 |
|  | p | 0.107 | 0.970 | 0.939 | 0.827 | 0.488 | 0.911 | 0.583 | 0.757 | 0.717 | **0.024** |
| **GLCM_Energy** | B | -0.001 | < - 0.001 | 0.002 | -0.001 | -0.012 | 0.002 | -0.009 | 0.000 | 0.003 | 0.030 |
|  | 95%CI | -0.003 | < - 0.001 | -0.003 | -0.004 | -0.024 | -0.017 | -0.032 | -0.002 | -0.009 | 0.015 |
|  |  | 0.000 | 0.001 | 0.007 | 0.001 | 0.000 | 0.021 | 0.015 | 0.002 | 0.015 | 0.046 |
|  | p | 0.079 | 0.688 | 0.456 | 0.272 | 0.056 | 0.819 | 0.463 | 0.887 | 0.594 | **<0.001** |
| **GLCM_Entropy_Log10** | B | 0.010 | 0.003 | -0.025 | 0.012 | 0.057 | -0.010 | 0.078 | -0.003 | -0.006 | -0.112 |
|  | 95%CI | 0.000 | -0.001 | -0.059 | -0.005 | -0.026 | -0.141 | -0.084 | -0.017 | -0.090 | -0.221 |
|  |  | 0.020 | 0.008 | 0.009 | 0.028 | 0.140 | 0.122 | 0.241 | 0.010 | 0.078 | -0.003 |
|  | p | **0.046** | 0.163 | 0.143 | 0.161 | 0.175 | 0.887 | 0.343 | 0.649 | 0.888 | **0.044** |
| **GLRLM_RLNU** | B | 213.100 | 45.000 | -429.700 | 213.500 | 1180.100 | 621.800 | 1526.400 | 452.600 | -2626.700 | -737.900 |
|  | 95%CI | 47.100 | -34.900 | -979.900 | -52.100 | -179.600 | -1537.700 | -1130.200 | 231.900 | -3996.100 | -2523.200 |
|  |  | 379.000 | 125.000 | 120.000 | 479.000 | 2540.000 | 2781.000 | 4183.000 | 673.000 | -1257.000 | 1047.000 |
|  | p | **0.012** | 0.270 | 0.126 | 0.115 | 0.089 | 0.573 | 0.260 | **< 0.001** | **< 0.001** | 0.418 |
| **NGLDM_Contrast** | B | 0.000 | 0.000 | -5.55e−4 | 0.000 | 0.003 | -0.001 | 0.000 | < - 0.001 | 0.002 | -0.004 |
|  | 95%CI | < - 0.001 | < - 0.001 | -0.002 | < - 0.001 | < - 0.001 | -0.006 | -0.005 | < - 0.001 | -0.001 | -0.008 |
|  |  | 0.000 | 0.000 | 0.001 | 0.001 | 0.006 | 0.003 | 0.006 | 0.000 | 0.005 | 0.000 |
|  | p | 0.641 | 0.879 | 0.356 | 0.869 | 0.055 | 0.594 | 0.904 | 0.100 | 0.199 | 0.053 |
| **GLZLM_SZE** | B | 0.003 | -3.85e−4 | -0.015 | 0.004 | 0.041 | -0.010 | 0.019 | -0.002 | -0.011 | -0.079 |
|  | 95%CI | -0.002 | -0.003 | -0.033 | -0.005 | -0.004 | -0.082 | -0.068 | -0.009 | -0.056 | -0.138 |
|  |  | 0.009 | 0.002 | 0.004 | 0.012 | 0.086 | 0.061 | 0.107 | 0.005 | 0.035 | -0.020 |
|  | p | 0.258 | 0.775 | 0.117 | 0.413 | 0.074 | 0.774 | 0.666 | 0.574 | 0.645 | **0.009** |
| **GLZLM_ZLNU** | B | 11.280 | 1.480 | -25.940 | 10.760 | 58.340 | 51.430 | 108.940 | 15.790 | -96.770 | -60.210 |
|  | 95%CI | 3.070 | -2.470 | -53.130 | -2.370 | -8.860 | -55.290 | -22.340 | 4.880 | -164.440 | -148.440 |
|  |  | 19.490 | 5.420 | 1.250 | 23.880 | 125.530 | 158.150 | 240.220 | 26.700 | -29.110 | 28.010 |
|  | p | **0.007** | 0.463 | 0.062 | 0.108 | 0.089 | 0.345 | 0.104 | **0.005** | **0.005** | 0.181 |
| **GLZLM_GLNU** | B | 6.347 | -0.027 | -11.988 | 8.298 | 56.056 | 37.275 | 56.971 | 19.028 | -105.767 | -68.037 |
|  | 95%CI | -0.086 | -3.121 | -33.302 | -1.992 | 3.380 | -46.381 | -45.942 | 10.476 | -158.812 | -137.197 |
|  |  | 12.780 | 3.070 | 9.330 | 18.590 | 108.730 | 120.930 | 159.880 | 27.580 | -52.720 | 1.120 |
|  | p | 0.053 | 0.986 | 0.270 | 0.114 | **0.037** | 0.382 | 0.278 | **< 0.001** | **< 0.001** | 0.054 |
