# Supplementary material for: Effect of Body Composition and Age on the Subjective and Quantitative Ultrasound Appearance of the Dogs’ Pancreas
Source: Vet Radiol Ultrasound. 2026 Jul 15;67(4):e70208. doi: 10.1111/vru.70208 (PMC13371151; doi:10.1111/vru.70208)
Supplement: Supplementary file 5 — vru70208‐Supp‐0005‐SuppMat5.docx [file VRU-67-0-s005.docx]

S5. Summary of Multivariate analysis of Quantitative Measures of Echotexture

1. **Multivariate Omnibus Testing of Quantitative Measures of Echotexture**

Table 1: Multivariate model evaluating associations between selected clinical predictors and pancreatic texture features in a cohort of 72 dogs

| Multivariate Tests | | | | | | |
| --- | --- | --- | --- | --- | --- | --- |
|  |  | **value** | **F** | **df1** | **df2** | **p** |
| **Probe_NOM** | **Pillai's Trace** | 0.3452 | 6.195 | 4 | 47 | **<0.001*** |
|  | **Wilks' Lambda** | 0.655 | 6.195 | 4 | 47 | **<0.001*** |
| **Orientation** | **Pillai's Trace** | 0.1314 | 1.778 | 4 | 47 | 0.149 |
|  | **Wilks' Lambda** | 0.869 | 1.778 | 4 | 47 | 0.149 |
| **Probe_NOM ✻ Orientation** | **Pillai's Trace** | 0.1055 | 1.386 | 4 | 47 | 0.253 |
|  | **Wilks' Lambda** | 0.894 | 1.386 | 4 | 47 | 0.253 |
| **Age (years)** | **Pillai's Trace** | 0.1343 | 1.822 | 4 | 47 | 0.140 |
|  | **Wilks' Lambda** | 0.866 | 1.822 | 4 | 47 | 0.140 |
| **Pancreas Thickness (mm)** | **Pillai's Trace** | 0.3681 | 6.845 | 4 | 47 | **<0.001*** |
|  | **Wilks' Lambda** | 0.632 | 6.845 | 4 | 47 | **<0.001*** |
| **VAT_Average (mm)** | **Pillai's Trace** | 0.0414 | 0.507 | 4 | 47 | 0.731 |
|  | **Wilks' Lambda** | 0.959 | 0.507 | 4 | 47 | 0.731 |

*statistically significant with p < 0.05

Table 2. Univariate analyses evaluating associations between selected clinical predictors and individual pancreatic texture features in 72 dogs

| Univariate Tests | | | | | | |
| --- | --- | --- | --- | --- | --- | --- |
|  | **Dependent Variable** | **Sum of Squares** | **df** | **Mean Square** | **F** | **p** |
| **Probe_NOM** | **CONVENTIONAL_std (3)** | 71.19911 | 1 | 71.19911 | 6.04559 | **0.017*** |
|  | **GLRLM_SRE (3)** | 0.17295 | 1 | 0.17295 | 18.29103 | **<0.001*** |
|  | **GLRLM_RLNU (3)** | 1.42e+7 | 1 | 1.42e+7 | 2.18933 | 0.145 |
|  | **NGLDM_Contrast (3)** | 5.19e-5 | 1 | 5.19e-5 | 1.30496 | 0.259 |
| **Orientation** | **CONVENTIONAL_std (3)** | 3.91540 | 1 | 3.91540 | 0.33246 | 0.567 |
|  | **GLRLM_SRE (3)** | 1.47e-4 | 1 | 1.47e-4 | 0.01560 | 0.901 |
|  | **GLRLM_RLNU (3)** | 4.14e+7 | 1 | 4.14e+7 | 6.38674 | 0.015 |
|  | **NGLDM_Contrast (3)** | 2.20e-5 | 1 | 2.20e-5 | 0.55259 | 0.461 |
| **Probe_NOM ✻ Orientation** | **CONVENTIONAL_std (3)** | 0.22858 | 1 | 0.22858 | 0.01941 | 0.890 |
|  | **GLRLM_SRE (3)** | 0.03884 | 1 | 0.03884 | 4.10722 | **0.048*** |
|  | **GLRLM_RLNU (3)** | 1.14e+6 | 1 | 1.14e+6 | 0.17543 | 0.677 |
|  | **NGLDM_Contrast (3)** | 6.07e-5 | 1 | 6.07e-5 | 1.52641 | 0.222 |
| **Age (years)** | **CONVENTIONAL_std (3)** | 60.61133 | 1 | 60.61133 | 5.14657 | **0.028*** |
|  | **GLRLM_SRE (3)** | 0.02305 | 1 | 0.02305 | 2.43758 | 0.125 |
|  | **GLRLM_RLNU (3)** | 3.68e+7 | 1 | 3.68e+7 | 5.68403 | **0.021*** |
|  | **NGLDM_Contrast (3)** | 8.66e-6 | 1 | 8.66e-6 | 0.21783 | 0.643 |
| **Pancreas Thickness (mm)** | **CONVENTIONAL_std (3)** | 0.21953 | 1 | 0.21953 | 0.01864 | 0.892 |
|  | **GLRLM_SRE (3)** | 9.19e-6 | 1 | 9.19e-6 | 9.72e-4 | 0.975 |
|  | **GLRLM_RLNU (3)** | 1.32e+8 | 1 | 1.32e+8 | 20.30244 | **<0.001*** |
|  | **NGLDM_Contrast (3)** | 3.71e-5 | 1 | 3.71e-5 | 0.93321 | 0.339 |
| **VAT_Average (mm)** | **CONVENTIONAL_std (3)** | 0.14067 | 1 | 0.14067 | 0.01194 | 0.913 |
|  | **GLRLM_SRE (3)** | 6.00e-5 | 1 | 6.00e-5 | 0.00634 | 0.937 |
|  | **GLRLM_RLNU (3)** | 1.03e+7 | 1 | 1.03e+7 | 1.58323 | 0.214 |
|  | **NGLDM_Contrast (3)** | 1.44e-5 | 1 | 1.44e-5 | 0.36189 | 0.550 |
| **Residuals** | **CONVENTIONAL_std (3)** | 588.85172 | 50 | 11.77703 |  |  |
|  | **GLRLM_SRE (3)** | 0.47277 | 50 | 0.00946 |  |  |
|  | **GLRLM_RLNU (3)** | 3.24e+8 | 50 | 6.48e+6 |  |  |
|  | **NGLDM_Contrast (3)** | 0.00199 | 50 | 3.98e-5 |  |  |

 *statistically significant with p < 0.05

1. **Principal Component Analysis of Quantitative Measures of Echotexture**

Table 3: Principal component loadings of pancreatic texture features in 72 dogs

| **Component Loadings** | | | |
| --- | --- | --- | --- |
|  | **Component** | |  |
|  | **PC1** | **PC2** | **Uniqueness** |
| **CONVENTIONAL_std (3)** | 0.812 |  | 0.2985 |
| **GLRLM_SRE (3)** | 0.459 | 0.804 | 0.1421 |
| **GLRLM_RLNU (3)** | 0.887 |  | 0.2067 |
| **NGLDM_Contrast (3)** |  | 0.944 | 0.0874 |
|  |  |  |  |
| **Percentage Variance (%)** | 49 | 33 |  |
| **Eigenvalues** | 1.96 | 1.30 |  |
| **Note. 'varimax' rotation was used** | | | |

 PC – principal component

1. **Multivariable Regression Modelling of PCA Components**

Table 4. Multivariable regression of the principal component texture score (PC1) on selected patient characteristics and ultrasound acquisition factors in 72 dogs

| **Name** | **Effect** | **Estimate** | **95% Confidence Intervals** | | **p** |
| --- | --- | --- | --- | --- | --- |
|  |  |  | **Lower** | **Upper** |  |
| (Intercept) | (Intercept) | -0.4418 | -0.7576 | -0.1261 | 0.006 |
| Age (years) | Age (years) | 0.0678 | 0.0125 | 0.1232 | **0.016*** |
| VAT_Average (mm) | VAT_Average (mm) | 0.0152 | -0.0177 | 0.0481 | 0.366 |
| Pancreas Thickness (mm) | Pancreas Thickness (mm) | 0.1013 | 0.0268 | 0.1758 | **0.008*** |
| Orientation1 | Transverse - Long | -0.6216 | -1.1210 | -0.1222 | **0.015*** |
| Probe_NOM1 | L18-5 - C8-5 | -0.5654 | -1.1747 | 0.0440 | 0.069 |

  *statistically significant with p < 0.05

Table 5. Sensitivity analysis of multivariable regression of the principal component texture score (PC1) on all patient characteristics and ultrasound acquisition factors in 72 dogs

| **Predictor** |  | **B** | **B 95% CI** | **p-value** |
| --- | --- | --- | --- | --- |
| Age (years) | BCS + complete VAT/SAT  (primary analysis) | 0.066 | 0.003, 0.129 | **0.041*** |
|  | BCS + imputed VAT/SAT | 0.054 | -0.003, 0.111 | 0.063 |
|  | BCS + complete VAT | 0.060 | -0.001, 0.122 | 0.055 |
|  | BCS + imputed VAT | 0.049 | -0.008, 0.105 | 0.090 |
|  | Complete TAT + complete VAT | 0.059 | -0.004, 0.122 | 0.066 |
|  | Imputed TAT + imputed VAT | 0.053 | -0.004, 0.111 | 0.070 |
| Weight (kg) | BCS + complete VAT/SAT  (primary analysis) | 0.006 | -0.026, 0.038 | 0.712 |
|  | BCS + imputed VAT/SAT | 0.001 | -0.025, 0.028 | 0.916 |
|  | BCS + complete VAT | -0.005 | -0.037, 0.027 | 0.764 |
|  | BCS + imputed VAT | -0.012 | -0.039, 0.016 | 0.410 |
|  | Complete TAT + complete VAT | -0.001 | -0.041, 0.039 | 0.954 |
|  | Imputed TAT + imputed VAT | -0.022 | -0.056, 0.012 | 0.210 |
| Total adiposity | BCS + complete VAT/SAT  (primary analysis) | -0.091 | -0.289, 0.106 | 0.364 |
|  | BCS + imputed VAT/SAT | -0.144 | -0.328, 0.040 | 0.124 |
|  | BCS + complete VAT | -0.107 | -0.313, 0.098 | 0.306 |
|  | BCS + imputed VAT | -0.166 | -0.349, 0.018 | 0.077 |
|  | Complete TAT + complete VAT | -0.042 | -0.182, 0.099 | 0.561 |
|  | Imputed TAT + imputed VAT | 0.035 | -0.109, 0.180 | 0.631 |
| Visceral adiposity | BCS + complete VAT/SAT  (primary analysis) | 0.067 | -0.029, 0.163 | 0.172 |
|  | BCS + imputed VAT/SAT | 0.074 | -0.007, 0.155 | 0.075 |
|  | BCS + complete VAT | 0.032 | -0.014, 0.079 | 0.173 |
|  | BCS + imputed VAT | 0.042 | 0.005, 0.079 | **0.026*** |
|  | Complete TAT + complete VAT | -0.005 | -0.142, 0.132 | 0.946 |
|  | Imputed TAT + imputed VAT | 0.061 | -0.080, 0.202 | 0.399 |
| Sex (Female vs Male) | BCS + complete VAT/SAT  (primary analysis) | 0.203 | -0.336, 0.743 | 0.460 |
|  | BCS + imputed VAT/SAT | 0.156 | -0.306, 0.618 | 0.507 |
|  | BCS + complete VAT | 0.250 | -0.277, 0.778 | 0.353 |
|  | BCS + imputed VAT | 0.217 | -0.237, 0.672 | 0.348 |
|  | Complete TAT + complete VAT | 0.209 | -0.376, 0.793 | 0.484 |
|  | Imputed TAT + imputed VAT | 0.357 | -0.131, 0.846 | 0.152 |
| Neuter status (Desexed vs entire) | BCS + complete VAT/SAT  (primary analysis) | 0.188 | -0.719, 1.095 | 0.684 |
|  | BCS + imputed VAT/SAT | 0.212 | -0.538, 0.962 | 0.579 |
|  | BCS + complete VAT | 0.240 | -0.684, 1.164 | 0.611 |
|  | BCS + imputed VAT | 0.324 | -0.430, 1.079 | 0.399 |
|  | Complete TAT + complete VAT | 0.226 | -0.709, 1.162 | 0.635 |
|  | Imputed TAT + imputed VAT | 0.441 | -0.334, 1.217 | 0.265 |
| HAC (Yes vs No) | BCS + complete VAT/SAT  (primary analysis) | 0.398 | -0.673, 1.469 | 0.467 |
|  | BCS + imputed VAT/SAT | 0.560 | -0.332, 1.453 | 0.219 |
|  | BCS + complete VAT | 0.498 | -0.549, 1.545 | 0.351 |
|  | BCS + imputed VAT | 0.596 | -0.280, 1.472 | 0.182 |
|  | Complete TAT + complete VAT | 0.295 | -0.698, 1.289 | 0.560 |
|  | Imputed TAT + imputed VAT | 0.347 | -0.503, 1.197 | 0.424 |
| Pancreas Thickness (mm) | BCS + complete VAT/SAT  (primary analysis) | 0.096 | 0.009, 0.183 | **0.030*** |
|  | BCS + imputed VAT/SAT | 0.098 | 0.022, 0.175 | **0.011*** |
|  | BCS + complete VAT | 0.101 | 0.015, 0.188 | **0.022*** |
|  | BCS + imputed VAT | 0.107 | 0.031, 0.183 | **0.006*** |
|  | Complete TAT + complete VAT | 0.100 | 0.011, 0.189 | **0.027*** |
|  | Imputed TAT + imputed VAT | 0.112 | 0.034, 0.190 | **0.005*** |
| Pancreas Orientation (Transverse - Longitudinal) | BCS + complete VAT/SAT  (primary analysis) | -0.663 | -1.204, -0.122 | **0.016*** |
|  | BCS + imputed VAT/SAT | -0.553 | -1.020, -0.086 | **0.020*** |
|  | BCS + complete VAT | -0.625 | -1.156, -0.094 | **0.021*** |
|  | BCS + imputed VAT | -0.565 | -1.025, -0.104 | **0.016*** |
|  | Complete TAT + complete VAT | -0.640 | -1.175, -0.105 | **0.019*** |
|  | Imputed TAT + imputed VAT | -0.601 | -1.076, -0.126 | **0.013*** |
| Transducer (C8-5 vs L18-5) | BCS + complete VAT/SAT  (primary analysis) | -0.655 | -1.309, -0.002 | **0.049*** |
|  | BCS + imputed VAT/SAT | -0.795 | -1.418, -0.171 | **0.013*** |
|  | BCS + complete VAT | -0.667 | -1.308, -0.025 | **0.042*** |
|  | BCS + imputed VAT | -0.797 | -1.410, -0.183 | **0.011*** |
|  | Complete TAT + complete VAT | -0.649 | -1.294, -0.004 | **0.048*** |
|  | Imputed TAT + imputed VAT | -0.755 | -1.385, -0.124 | **0.019*** |

*statistically significant with p < 0.05

Model: principal component of echotexture = intercept + age + weight + substituted adiposity (BCS, complete-TAT, imputed-TAT) + substituted fat distribution (complete-VAT/SAT, imputed-VAT/SAT, complete-VAT, imputed-VAT) + sex + neuter status + HAC diagnosis + pancreatic thickness + image orientation + transducer frequency.
