# Supplementary material for: Effect of Body Composition and Age on the Subjective and Quantitative Ultrasound Appearance of the Dogs’ Pancreas
Source: Vet Radiol Ultrasound. 2026 Jul 15;67(4):e70208. doi: 10.1111/vru.70208 (PMC13371151; doi:10.1111/vru.70208)
Supplement: Supplementary file 6 — vru70208‐Supp‐0005‐SuppMat6.docx [file VRU-67-0-s002.docx]

S4. Validation of Quantitative Measures of Echogenicity and Echotexture

Texture variables that distinguished subjective echotexture (homogeneous vs heterogeneous) underwent discrimination, redundancy, and stability analyses to reduce multicollinearity and preserve interpretability of the principal component analysis (PCA).

Discrimination of heterogeneous vs homogeneous echotexture was assessed using the Mann–Whitney U test with rank-biserial effect size (table 1) and receiver operator curve (ROC) area under the curve (AUC) (95% CI) (table 3).

Correlation of variables are summarised in table 2. Texture variables with Pearson’s correlations exceeding |0.80| were deemed redundant and a single representative variable used for analysis.

Sensitivity analysis selected final variables if comparable discretisation (bins 10/d1, bins 10/d10, and bins 5/d5) had differences AUC (|ΔAUC|) less than 0.05 and a non-significant paired DeLong test (19).

The general linear model analysis of univariate quantitative echotexture features and associated predictors is presented in Table 4.

Table 1: Group discrimination of homogeneous and heterogeneous pancreatic echotexture using quantitative texture features (Mann-Whitney U Test)

|  | **Statistic** | **p** |  | **Effect Size** |
| --- | --- | --- | --- | --- |
| **CONVENTIONAL_std (3)** | 224 | **<.001** | Rank biserial correlation | 0.6475 |
| **DISCRETIZED_std (3)** | 262 | **<.001** | Rank biserial correlation | 0.5877 |
| **DISCRETIZED_HISTO_Entropy_log10 (3)** | 292 | **<.001** | Rank biserial correlation | 0.5405 |
| **DISCRETIZED_HISTO_Energy[=Uniformity] (3)** | 293 | **<.001** | Rank biserial correlation | -0.5389 |
| **GLCM_Homogeneity[=InverseDifference] (3)** | 498 | 0.120 | Rank biserial correlation | -0.2164 |
| **GLCM_Energy[=AngularSecondMoment] (3)** | 364 | **0.002** | Rank biserial correlation | -0.4272 |
| **GLCM_Contrast[=Variance] (3)** | 458 | **0.044** | Rank biserial correlation | 0.2793 |
| **GLCM_Correlation (3)** | 402 | **0.007** | Rank biserial correlation | 0.3674 |
| **GLCM_Entropy_log10 (3)** | 341 | **<.001** | Rank biserial correlation | 0.4634 |
| **GLCM_Dissimilarity (3)** | 487 | 0.092 | Rank biserial correlation | 0.2337 |
| **GLRLM_SRE (3)** | 459 | **0.045** | Rank biserial correlation | 0.2777 |
| **GLRLM_LRE (3)** | 473 | 0.065 | Rank biserial correlation | -0.2557 |
| **GLRLM_LGRE (3)** | 627 | 0.928 | Rank biserial correlation | 0.0134 |
| **GLRLM_HGRE (3)** | 570 | 0.462 | Rank biserial correlation | 0.1031 |
| **GLRLM_SRLGE (3)** | 599 | 0.684 | Rank biserial correlation | 0.0574 |
| **GLRLM_SRHGE (3)** | 507 | 0.146 | Rank biserial correlation | 0.2022 |
| **GLRLM_LRLGE (3)** | 578 | 0.519 | Rank biserial correlation | -0.0905 |
| **GLRLM_LRHGE (3)** | 592 | 0.627 | Rank biserial correlation | -0.0685 |
| **GLRLM_GLNU (3)** | 515 | 0.173 | Rank biserial correlation | 0.1896 |
| **GLRLM_RLNU (3)** | 355 | **0.001** | Rank biserial correlation | 0.4414 |
| **GLRLM_RP (3)** | 491 | 0.102 | Rank biserial correlation | 0.2274 |
| **NGLDM_Coarseness (3)** | 544 | 0.303 | Rank biserial correlation | -0.1440 |
| **NGLDM_Contrast (3)** | 344 | **<.001** | Rank biserial correlation | 0.4587 |
| **NGLDM_Busyness (3)** | 575 | 0.497 | Rank biserial correlation | 0.0952 |
| **GLZLM_SZE (3)** | 430 | **0.019** | Rank biserial correlation | 0.3234 |
| **GLZLM_LZE (3)** | 505 | 0.140 | Rank biserial correlation | -0.2054 |
| **GLZLM_LGZE (3)** | 596 | 0.659 | Rank biserial correlation | -0.0622 |
| **GLZLM_HGZE (3)** | 538 | 0.272 | Rank biserial correlation | 0.1534 |
| **GLZLM_SZLGE (3)** | 606 | 0.743 | Rank biserial correlation | 0.0464 |
| **GLZLM_SZHGE (3)** | 443 | **0.028** | Rank biserial correlation | 0.3029 |
| **GLZLM_LZLGE (3)** | 549 | 0.330 | Rank biserial correlation | -0.1361 |
| **GLZLM_LZHGE (3)** | 513 | 0.166 | Rank biserial correlation | -0.1928 |
| **GLZLM_GLNU (3)** | 434 | **0.022** | Rank biserial correlation | 0.3171 |
| **GLZLM_ZLNU (3)** | 379 | **0.003** | Rank biserial correlation | 0.4036 |
| **GLZLM_ZP (3)** | 467 | 0.056 | Rank biserial correlation | 0.2651 |

 * statistically significant with p < 0.05 were included in redundancy analysis

Table 2. Pearsons (*r)* correlation between significant textural features discriminating pancreas heterogeneity to assess for redundancy of variable

|  | **CONVENTIONAL_std (3)** | **DISCRETIZED_std (3)** | **DISCRETIZED_HISTO_Entropy_log10 (3)** | **DISCRETIZED_HISTO_Energy[=Uniformity] (3)** | **GLCM_Energy[=AngularSecondMoment] (3)** | **GLCM_Contrast[=Variance] (3)** | **GLCM_Correlation (3)** | **GLCM_Entropy_log10 (3)** | **GLRLM_SRE (3)** | **GLRLM_RLNU (3)** | **NGLDM_Contrast (3)** | **GLZLM_SZE (3)** | **GLZLM_SZHGE (3)** | **GLZLM_GLNU (3)** | **GLZLM_ZLNU (3)** |
| --- | --- | --- | --- | --- | --- | --- | --- | --- | --- | --- | --- | --- | --- | --- | --- |
| **CONVENTIONAL_std (3)** | — |  |  |  |  |  |  |  |  |  |  |  |  |  |  |
| **DISCRETIZED_std (3)** | 0.870 | — |  |  |  |  |  |  |  |  |  |  |  |  |  |
| **DISCRETIZED_HISTO_Entropy_log10 (3)** | 0.897 | 0.959 | — |  |  |  |  |  |  |  |  |  |  |  |  |
| **DISCRETIZED_HISTO_Energy[=Uniformity] (3)** | -0.870 | -0.897 | -0.978 | — |  |  |  |  |  |  |  |  |  |  |  |
| **GLCM_Energy[=AngularSecondMoment] (3)** | -0.705 | -0.783 | -0.841 | 0.829 | — |  |  |  |  |  |  |  |  |  |  |
| **GLCM_Contrast[=Variance] (3)** | -0.026 | 0.442 | 0.324 | -0.242 | -0.418 | — |  |  |  |  |  |  |  |  |  |
| **GLCM_Correlation (3)** | 0.325 | 0.060 | 0.144 | -0.232 | 0.203 | -0.677 | — |  |  |  |  |  |  |  |  |
| **GLCM_Entropy_log10 (3)** | 0.659 | 0.857 | 0.853 | -0.797 | -0.944 | 0.643 | -0.321 | — |  |  |  |  |  |  |  |
| **GLRLM_SRE (3)** | 0.432 | 0.398 | 0.427 | -0.382 | -0.799 | 0.239 | -0.442 | 0.713 | — |  |  |  |  |  |  |
| **GLRLM_RLNU (3)** | 0.486 | 0.607 | 0.582 | -0.549 | -0.568 | 0.316 | 0.053 | 0.599 | 0.348 | — |  |  |  |  |  |
| **NGLDM_Contrast (3)** | 0.080 | 0.040 | 0.067 | -0.046 | -0.344 | 0.237 | -0.643 | 0.333 | 0.595 | -0.146 | — |  |  |  |  |
| **GLZLM_SZE (3)** | 0.245 | 0.254 | 0.219 | -0.146 | -0.577 | 0.326 | -0.671 | 0.556 | 0.858 | 0.177 | 0.713 | — |  |  |  |
| **GLZLM_SZHGE (3)** | 0.428 | 0.527 | 0.506 | -0.392 | -0.626 | 0.431 | -0.495 | 0.693 | 0.655 | 0.354 | 0.467 | 0.694 | — |  |  |
| **GLZLM_GLNU (3)** | 0.449 | 0.438 | 0.450 | -0.444 | -0.502 | 0.073 | 0.139 | 0.465 | 0.416 | 0.937 | -0.104 | 0.233 | 0.289 | — |  |
| **GLZLM_ZLNU (3)** | 0.597 | 0.635 | 0.614 | -0.561 | -0.621 | 0.238 | 0.006 | 0.646 | 0.508 | 0.900 | 0.046 | 0.391 | 0.536 | 0.916 | — |

Correlations exceeding |0.80| were deemed redundant and a single representative variable used for sensitivity analysis

Table 3: Significant Echotexture Features Discriminating Pancreas Heterogeneity Identified by Mann-Whitney U Test after False Discovery Rate Correction (Benjamini-Hochberg, 5% Significance; n=72; Bin10/d1) with Sensitivity Across Settings (AUC) for Paired Analysis (n=48)

|  | **Discrimination** | | | | **Sensitivity Analysis** | | | | |
| --- | --- | --- | --- | --- | --- | --- | --- | --- | --- |
| **Feature** | **Non-adjusted p-value** | **FDR**  **q-value** | **r_rb_** | **bin10/d1**  **AUC (s.e.)** | **bin10/d1**  **AUC (s.e.)** | **bin10/d10**  **AUC (s.e.)** | **bin5/d5**  **AUC (s.e.)** | **Max**  **\|Δ AUC\|** | **DeLong Test**  **Min p value** |
| Number of cases | 74 | | | | 48 | 48 | 48 |  |  |
| CONVENTIONAL_std (3) | <0.001 | **0.002*** | 0.648 | 0.824 (0.047) | 0.880 (0.048) | 0.880 (0.048) | 0.880 (0.048) | **0.000^#^** | **1.000^#^** |
| GLCM_Contrast (3) | 0.044 | **0.045*** | 0.279 | 0.640 (0.066) | 0.713 (0.075) | 0.839 (0.059) | 0.774 (0.067) | 0.126 | 0.011 |
| GLCM_Correlation (3) | 0.007 | **0.011*** | 0.367 | 0.684 (0.066) | 0.676 (0.080) | 0.515 (0.084) | 0.596 (0.083) | 0.161 | 0.011 |
| GLCM_Entropy_log10 (3) | <0.001 | **0.002*** | 0.463 | 0.732 (0.059) | 0.776 (0.068) | 0.870 (0.052) | 0.841 (0.057) | 0.094 | 0.024 |
| GLRLM_SRE (3) | 0.045 | **0.045*** | 0.278 | 0.639 (0.067) | 0.700 (0.079) | 0.693 (0.078) | 0.689 (0.078) | **0.011^#^** | **0.598^#^** |
| GLRLM_RLNU (3) | 0.001 | **0.002*** | 0.441 | 0.721 (0.061) | 0.776 (0.067) | 0.807 (0.062) | 0.785 (0.068) | **0.031^#^** | **0.289^#^** |
| NGLDM_Contrast (3) | <0.001 | **0.002*** | 0.459 | 0.729 (0.060) | 0.717 (0.078) | 0.691 (0.079) | 0.722 (0.075) | **0.031^#^** | **0.212^#^** |
| GLZLM_SZHGE (3) | 0.028 | **0.037*** | 0.303 | 0.651 (0.066) | 0.735 (0.075) | 0.761 (0.072) | 0.709 (0.079) | 0.052 | 0.188 |

AUC – Area under the curve; FDR - False Discovery Rate; r_rb_ - rank-biserial.

*statistically significant with p < 0.05.

**^#^**Sensitivity analysis selected final variables if comparable discretisation (bins 10/d1, bins 10/d10, and bins 5/d5) had differences AUC (|ΔAUC|) less than 0.05 and a non-significant paired DeLong test.

Table 4. General linear model analysis of univariate quantitative echotexture features and associated predictors

|  |  | **Age (yr)** | **Weight (kg)** | **BCS** | **VAT/SAT** | **SEX** | **Neuter Status** | **HAC** | **Pancreas Thickness (mm)** | **Orientation** | **Probe** |
| --- | --- | --- | --- | --- | --- | --- | --- | --- | --- | --- | --- |
| **Conventional_std** | B | 0.174 | -0.031 | -0.299 | 0.058 | 0.126 | 0.141 | 1.709 | 0.007 | -0.209 | -4.040 |
|  | 95%CI | -0.048 | -0.137 | -1.033 | -0.297 | -1.690 | -2.743 | -1.839 | -0.288 | -2.038 | -6.424 |
|  |  | 0.396 | 0.076 | 0.436 | 0.413 | 1.942 | 3.025 | 5.256 | 0.302 | 1.619 | -1.656 |
|  | p | 0.125 | 0.572 | 0.426 | 0.749 | 0.892 | 0.924 | 0.345 | 0.962 | 0.822 | **<0.001*** |
| **Discretised_std** | B | 0.027 | 0.007 | -0.056 | 0.018 | 0.019 | 0.020 | 0.194 | -0.006 | -0.034 | -0.212 |
|  | 95%CI | 0.001 | -0.005 | -0.141 | -0.023 | -0.190 | -0.313 | -0.215 | -0.040 | -0.244 | -0.486 |
|  |  | 0.053 | 0.020 | 0.029 | 0.059 | 0.229 | 0.352 | 0.603 | 0.028 | 0.177 | 0.063 |
|  | p | **0.039*** | 0.243 | 0.194 | 0.397 | 0.856 | 0.908 | 0.352 | 0.713 | 0.754 | 0.131 |
| **Discretised_Entropy_Log10** | B | 0.005 | 0.001 | -0.005 | 0.002 | 0.010 | 0.002 | 0.039 | 0.000 | -0.004 | -0.056 |
|  | 95%CI | < - 0.001 | -0.002 | -0.023 | -0.006 | -0.034 | -0.068 | -0.047 | -0.007 | -0.049 | -0.113 |
|  |  | 0.010 | 0.003 | 0.012 | 0.011 | 0.053 | 0.071 | 0.124 | 0.007 | 0.040 | 0.002 |
|  | p | 0.067 | 0.640 | 0.545 | 0.595 | 0.667 | 0.966 | 0.375 | 0.930 | 0.842 | 0.056 |
| **Discretised_Energy** | B | -0.002 | < - 0.001 | < - 0.001 | < - 0.001 | -0.006 | -0.002 | -0.009 | < - 0.001 | 0.003 | 0.026 |
|  | 95%CI | -0.004 | -0.001 | -0.007 | -0.004 | -0.023 | -0.029 | -0.043 | -0.003 | -0.014 | 0.003 |
|  |  | 0.000 | 0.001 | 0.007 | 0.003 | 0.011 | 0.026 | 0.024 | 0.002 | 0.021 | 0.049 |
|  | p | 0.107 | 0.970 | 0.939 | 0.827 | 0.488 | 0.911 | 0.583 | 0.757 | 0.717 | **0.024*** |
| **GLCM_Energy** | B | -0.001 | < - 0.001 | 0.002 | -0.001 | -0.012 | 0.002 | -0.009 | 0.000 | 0.003 | 0.030 |
|  | 95%CI | -0.003 | < - 0.001 | -0.003 | -0.004 | -0.024 | -0.017 | -0.032 | -0.002 | -0.009 | 0.015 |
|  |  | 0.000 | 0.001 | 0.007 | 0.001 | 0.000 | 0.021 | 0.015 | 0.002 | 0.015 | 0.046 |
|  | p | 0.079 | 0.688 | 0.456 | 0.272 | 0.056 | 0.819 | 0.463 | 0.887 | 0.594 | **<0.001*** |
| **GLCM_Entropy_Log10** | B | 0.010 | 0.003 | -0.025 | 0.012 | 0.057 | -0.010 | 0.078 | -0.003 | -0.006 | -0.112 |
|  | 95%CI | 0.000 | -0.001 | -0.059 | -0.005 | -0.026 | -0.141 | -0.084 | -0.017 | -0.090 | -0.221 |
|  |  | 0.020 | 0.008 | 0.009 | 0.028 | 0.140 | 0.122 | 0.241 | 0.010 | 0.078 | -0.003 |
|  | p | **0.046*** | 0.163 | 0.143 | 0.161 | 0.175 | 0.887 | 0.343 | 0.649 | 0.888 | **0.044*** |
| **GLRLM_RLNU** | B | 213.100 | 45.000 | -429.700 | 213.500 | 1180.100 | 621.800 | 1526.400 | 452.600 | -2626.700 | -737.900 |
|  | 95%CI | 47.100 | -34.900 | -979.900 | -52.100 | -179.600 | -1537.700 | -1130.200 | 231.900 | -3996.100 | -2523.200 |
|  |  | 379.000 | 125.000 | 120.000 | 479.000 | 2540.000 | 2781.000 | 4183.000 | 673.000 | -1257.000 | 1047.000 |
|  | p | **0.012*** | 0.270 | 0.126 | 0.115 | 0.089 | 0.573 | 0.260 | **< 0.001*** | **< 0.001*** | 0.418 |
| **NGLDM_Contrast** | B | 0.000 | 0.000 | -5.55e−4 | 0.000 | 0.003 | -0.001 | 0.000 | < - 0.001 | 0.002 | -0.004 |
|  | 95%CI | < - 0.001 | < - 0.001 | -0.002 | < - 0.001 | < - 0.001 | -0.006 | -0.005 | < - 0.001 | -0.001 | -0.008 |
|  |  | 0.000 | 0.000 | 0.001 | 0.001 | 0.006 | 0.003 | 0.006 | 0.000 | 0.005 | 0.000 |
|  | p | 0.641 | 0.879 | 0.356 | 0.869 | 0.055 | 0.594 | 0.904 | 0.100 | 0.199 | 0.053 |
| **GLZLM_SZE** | B | 0.003 | -3.85e−4 | -0.015 | 0.004 | 0.041 | -0.010 | 0.019 | -0.002 | -0.011 | -0.079 |
|  | 95%CI | -0.002 | -0.003 | -0.033 | -0.005 | -0.004 | -0.082 | -0.068 | -0.009 | -0.056 | -0.138 |
|  |  | 0.009 | 0.002 | 0.004 | 0.012 | 0.086 | 0.061 | 0.107 | 0.005 | 0.035 | -0.020 |
|  | p | 0.258 | 0.775 | 0.117 | 0.413 | 0.074 | 0.774 | 0.666 | 0.574 | 0.645 | **0.009*** |
| **GLZLM_ZLNU** | B | 11.280 | 1.480 | -25.940 | 10.760 | 58.340 | 51.430 | 108.940 | 15.790 | -96.770 | -60.210 |
|  | 95%CI | 3.070 | -2.470 | -53.130 | -2.370 | -8.860 | -55.290 | -22.340 | 4.880 | -164.440 | -148.440 |
|  |  | 19.490 | 5.420 | 1.250 | 23.880 | 125.530 | 158.150 | 240.220 | 26.700 | -29.110 | 28.010 |
|  | p | **0.007*** | 0.463 | 0.062 | 0.108 | 0.089 | 0.345 | 0.104 | **0.005*** | **0.005*** | 0.181 |
| **GLZLM_GLNU** | B | 6.347 | -0.027 | -11.988 | 8.298 | 56.056 | 37.275 | 56.971 | 19.028 | -105.767 | -68.037 |
|  | 95%CI | -0.086 | -3.121 | -33.302 | -1.992 | 3.380 | -46.381 | -45.942 | 10.476 | -158.812 | -137.197 |
|  |  | 12.780 | 3.070 | 9.330 | 18.590 | 108.730 | 120.930 | 159.880 | 27.580 | -52.720 | 1.120 |
|  | p | 0.053 | 0.986 | 0.270 | 0.114 | **0.037*** | 0.382 | 0.278 | **< 0.001*** | **< 0.001*** | 0.054 |

*statistically significant with p < 0.05.
